# Supplementary figures and images for: A Foxf1-Wnt-Nr2f1 cascade promotes atrial cardiomyocyte differentiation in zebrafish
Source: PLoS Genet. 2024 Nov 4;20(11):e1011222. doi: 10.1371/journal.pgen.1011222 (PMC11563408; doi:10.1371/journal.pgen.1011222)

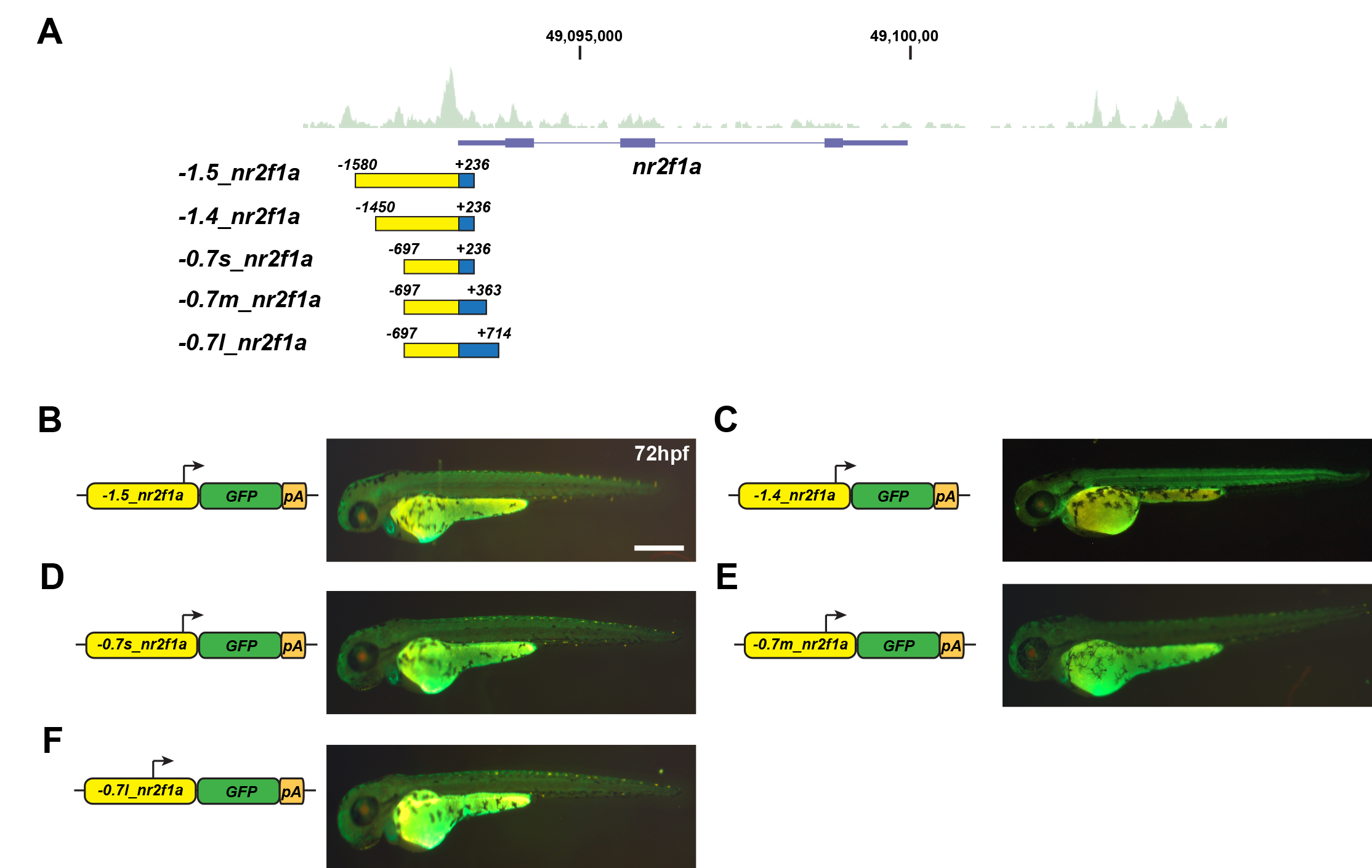

Supplement: S1 Fig — A) Schematic of open chromatin in ACs from ATAC-seq showing the putative promoter fragments relative to the nr2f1a locus that were analyzed with stable transgenic lines. Image of tracks generated in UCSC genome browser (https://genome.ucsc.edu). B-F) Schematics of the nr2f1a promoter transgenic constructs and images of representative stable transgenic lines. All the promoter constructs showed broad expression throughout the embryos. Lateral view with anterior left and dorsal upward. Scale bar: 500 μm. (TIF) [file pgen.1011222.s001.tif]

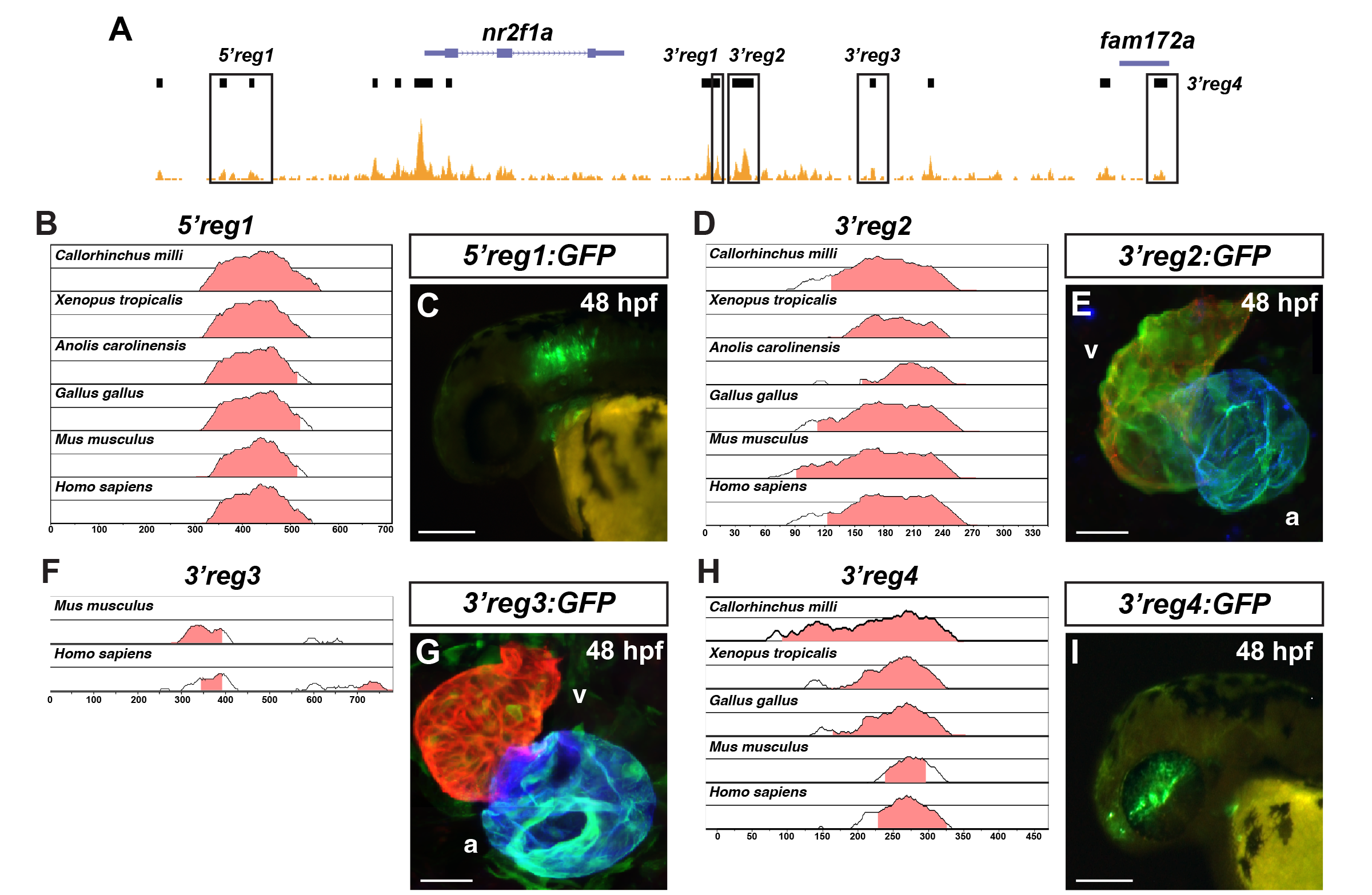

Supplement: S2 Fig — A) Schematic of ATAC-seq data in ACs showing the localization of additional putative enhancers that were examined with reporters relative to the nr2f1a locus. B) VISTA plot showing conservation of the zebrafish 5’reg1-nr2f1a enhancers with regions in Callorhinchus milli (Australian ghostshark), Xenopus tropicalis (Tropical clawed frog), Anolis carolinensis (Green Anole), Gallus gallus (chicken), Mus musculus (House mouse), and Homo sapiens (human). C) Image of 5’reg1:GFP embryo with expression in the anterior hindbrain and branchial arches at 48 hpf. Lateral view is anterior to the left and dorsal upward. (n = 25). Scale bar: 200 μm. D) VISTA plot showing conservation of the zebrafish 3’reg2-nr2f1a enhancer with regions in Callorhinchus milli (Australian ghostshark), Xenopus tropicalis (Tropicalis clawed frog), Anolis carolinensis (green Anole), Gallus gallus (chicken), Mus musculus (House mouse), and Homo sapiens (human). E) Confocal image of heart at 48 hpf from 3’reg2:GFP embryo stained for 3’reg2:GFP (green), Vmhc (red), and Amhc (blue). V indicates ventricle. A indicates atrium. (n = 21). Scale bar: 50 μm. F) VISTA plot showing conservation of the zebrafish 3’reg3-nr2f1a enhancer with regions in Mus musculus (House mouse) and Homo sapiens (human). G) Confocal images of heart at 48 hpf from 3’reg3:GFP embryos stained for 3’reg3:GFP (green), Vmhc (red), and Amhc (blue). V indicates ventricle. A indicates atrium. Scale bar: 50 μm. H) VISTA plot showing conservation of the zebrafish 3’reg4-nr2f1a enhancer with regions in Callorhinchus milli (Australian ghostshark), Xenopus tropicalis (Tropical clawed frog), Gallus gallus (chicken), Mus musculus (House mouse), and Homo sapiens (human). I) Image of the 3’reg4:GFP embryo with expression in the medial eye, the anterior brain, the nasal pits, and the first branchial arch at 48 hpf (n = 23). Scale bar: 200 μm. Pink in VISTA plots indicates >50% conservation of regulatory regions with the zebrafish enhancer sequence. Medi [file pgen.1011222.s002.tif]

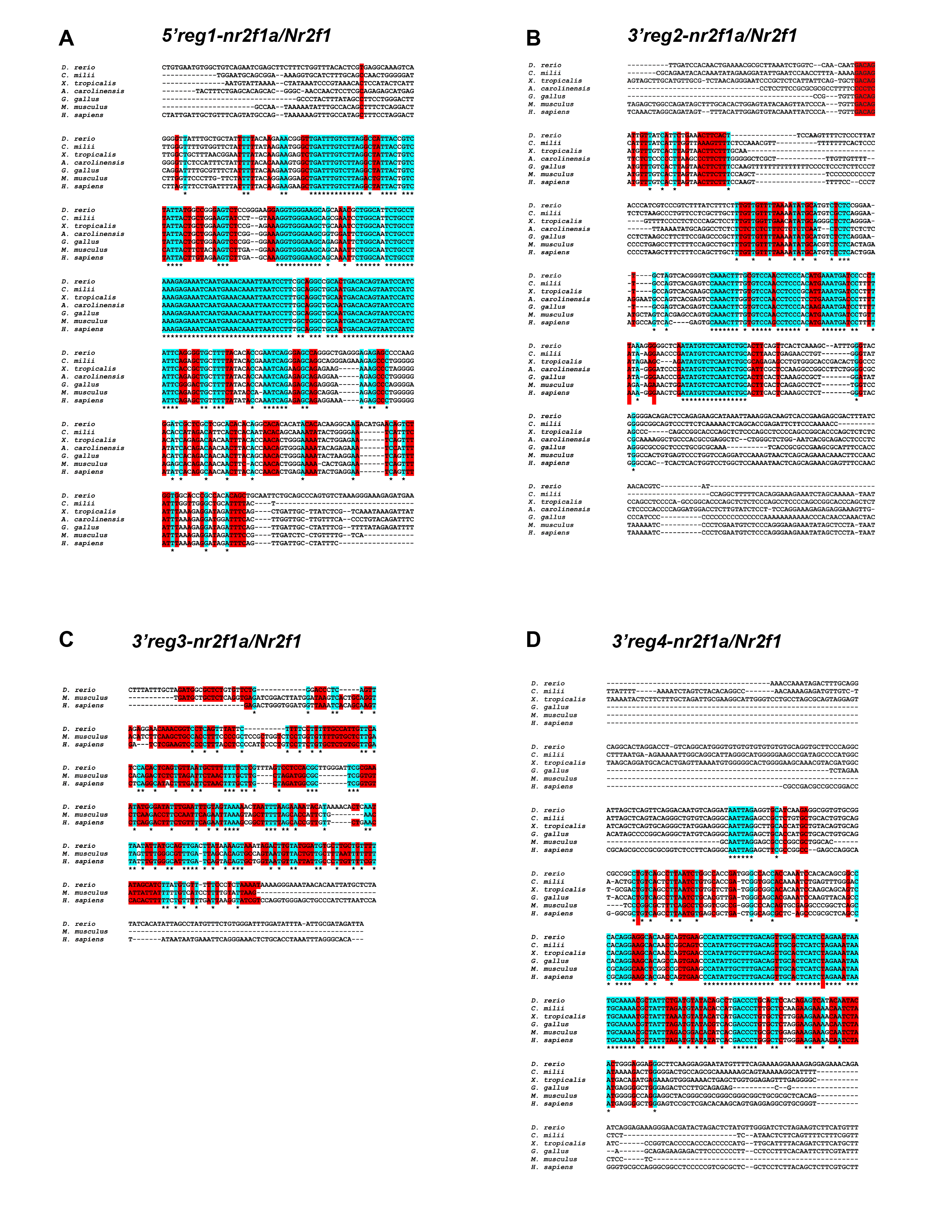

Supplement: S3 Fig — A-D) Clustal alignments of 5’reg1-nr2f1a/Nr2f1, 3’reg2-nr2f1a/Nr2f1, 3’reg3-nr2f1a/Nr2f1, 3’reg4-nr2f1a/Nr2f1 between zebrafish and additional vertebrate species: Callorhinchus milli (Australian ghostshark), Xenopus tropicalis (Tropical clawed frog), Gallus gallus (chicken), Anolis carolinensis (Green Anole), Mus musculus (House mouse), and Homo sapiens (human). Turquoise indicates completely conserved nucleotides. Red indicates partially conserved nucleotides. (TIF) [file pgen.1011222.s003.tif]

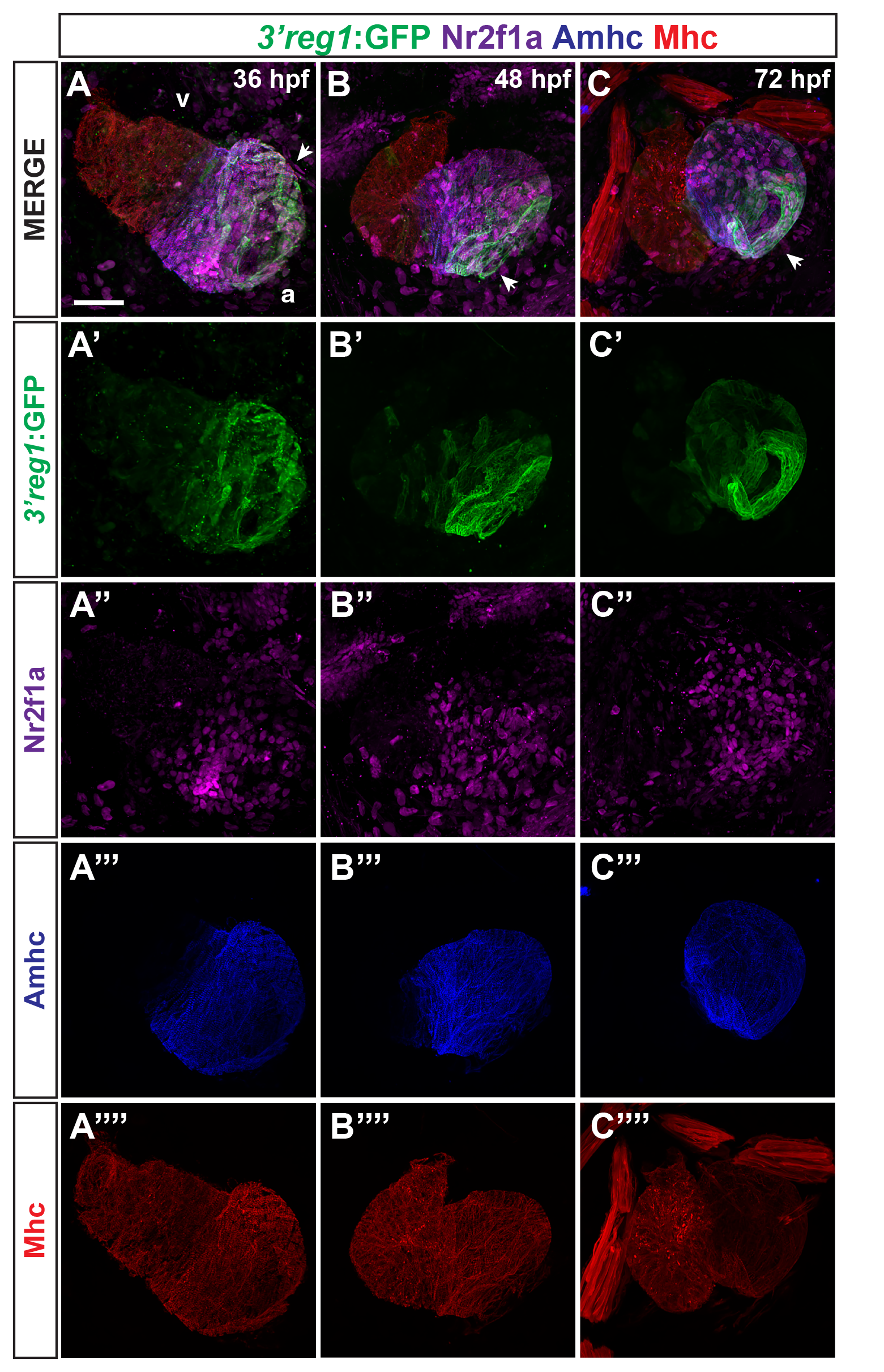

Supplement: S4 Fig — A-C”“) Merged and individual channels of the confocal images of hearts from Fig 1F–1H. Hearts from transgenic 3’reg1:GFP embryos stained for (A’-C’) 3’reg1:GFP (green), (A”-C”) Nr2f1a (magenta), (A”’-C”’) Amhc (ACs–blue), and (A”“-C”“) Mhc (pan-cardiac–red). 36 hpf (n = 4), 48 hpf (n = 4), 72 hpf (n = 4). v–ventricle. a–atrium. 3’reg1:GFP in the atria of the hearts (white arrowheads). Images are frontal views with the arterial pole up. n indicates the number of embryos examined for representative experiment. Scale bar: 50 μm. (TIF) [file pgen.1011222.s004.tif]

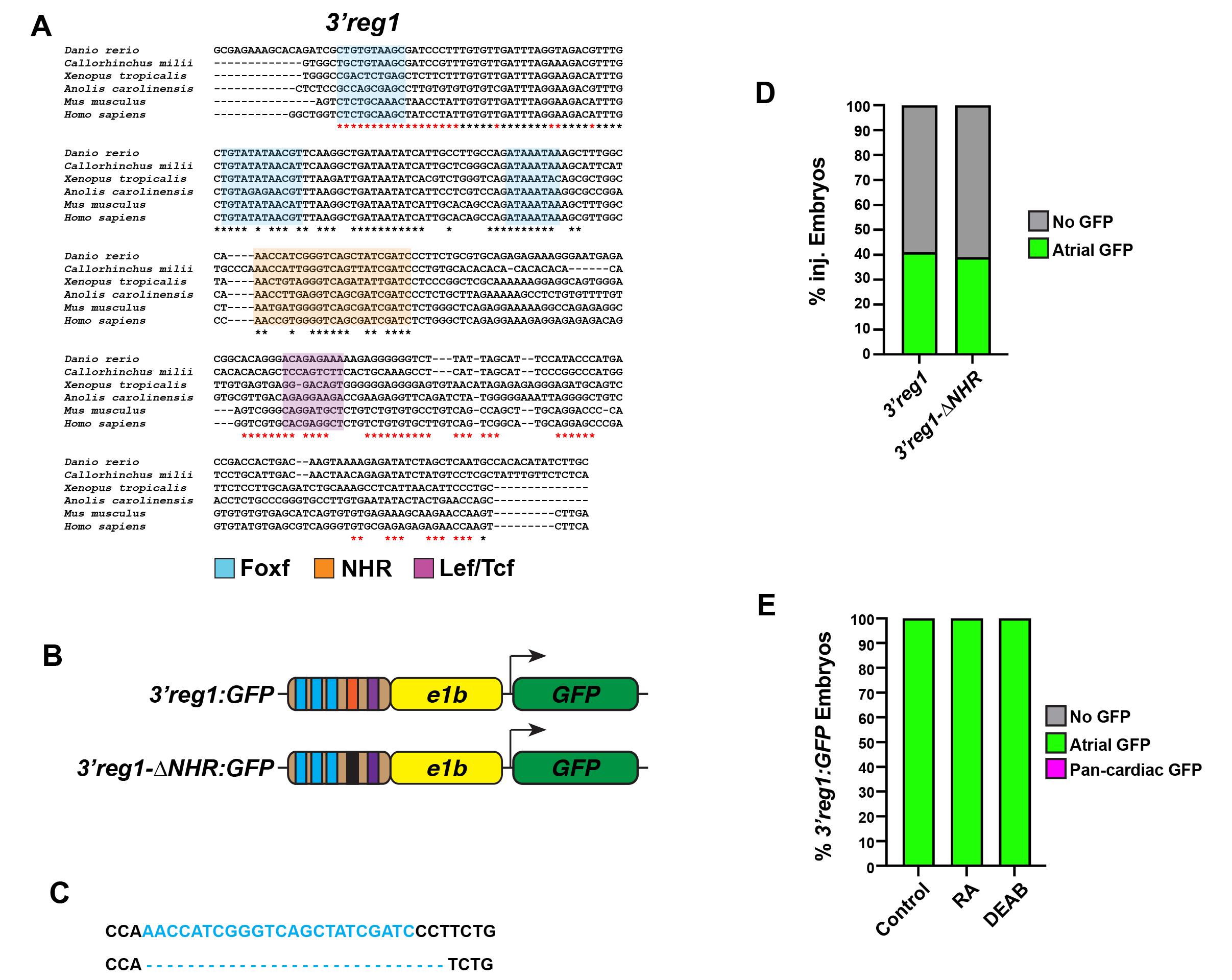

Supplement: S5 Fig — A) Clustal alignment of 3’reg1 with the putative NHR site highlighted, as well as Foxf1 and Lef/Tcf sites as shown in Fig 1. B) Schematics of 3’reg1:GFP reporter constructs. Foxf sites (blue), Lef/Tcf site (purple), NHR site (orange), deleted NHR site (black). C) Sequences showing WT NHR site and deletion of the NHR site in the 3’reg1:GFP constructs. Deletion of the NHR site did not affect expression within the heart relative to the WT 3’reg1:GFP construct (n = 48) nor did treatment with the RA signaling inhibitor DEAB (N = 33). D) The percentage of transient transgenic embryos with reporter expression in the atria of their hearts. 3’reg1:GFP (n = 21); 3’reg1-ΔNHR:GFP (n = 26). E) The percentage of stable 3’reg1:GFP embryos with no, atrial, and pan-cardiac reporter expression in their hearts follow treatment with RA or the RA signaling inhibitor DEAB. Control (n = 19); RA (n = 25); DEAB (n = 26). (TIF) [file pgen.1011222.s005.tif]

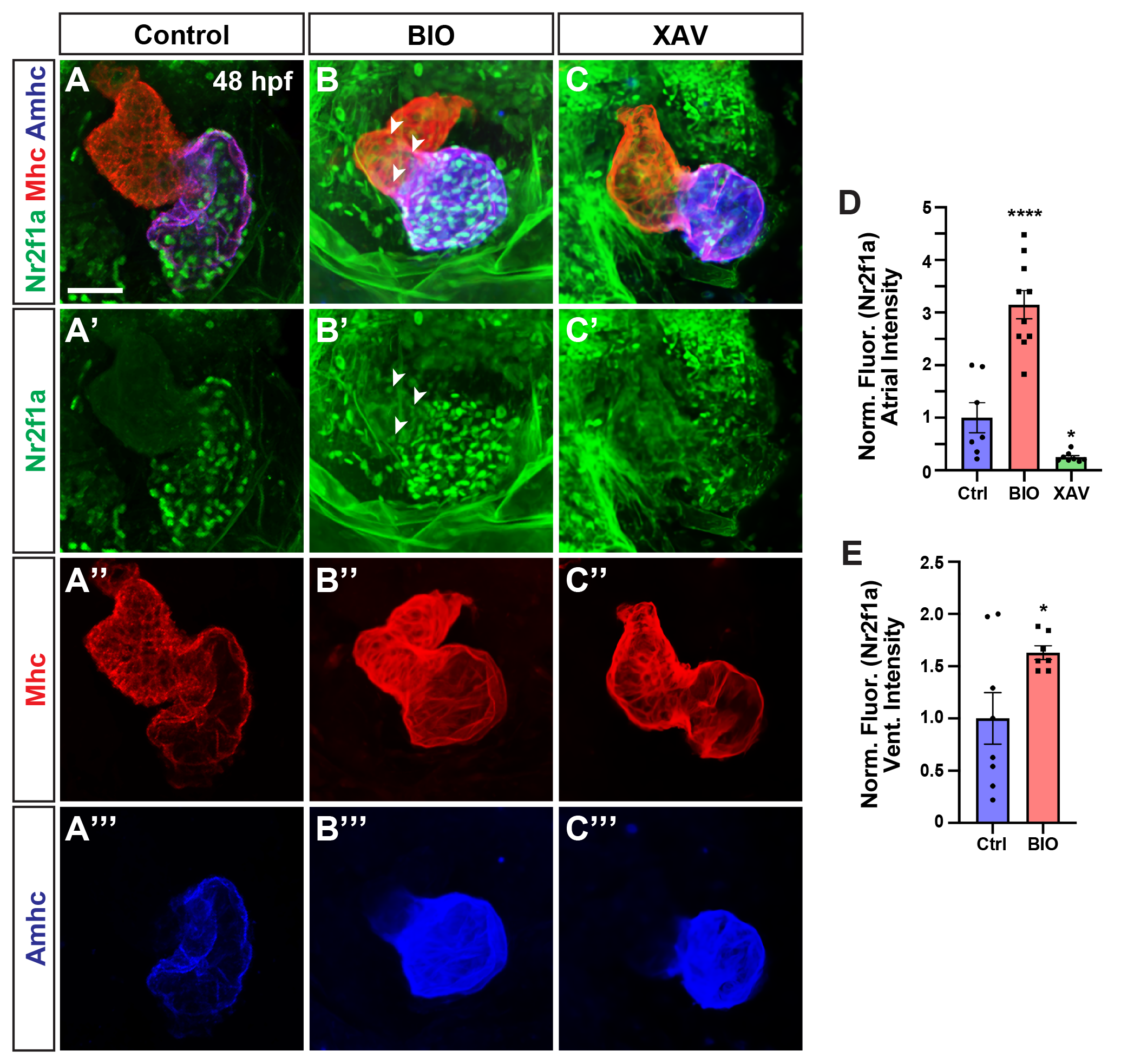

Supplement: S6 Fig — A-C”“) Merged and individual channels of the confocal images of hearts from Fig 4F–4H. Hearts from transgenic 3’reg1:GFP embryos stained for (A’-C’) Nr2f1a (green), (A”-C”) Mhc (pan-cardiac–red) (magenta), and (A”’-C”’) Amhc (ACs–blue). VCs with a low level of Nr2f1a within the nuclei (white arrowheads). Images are frontal views with the arterial pole up. Scale bar: 50 μm. D) Normalized intensity of Nr2f1a staining in atria of hearts from control, BIO-, and XAV-treated embryos. Control (n = 7); BIO (n = 10); XAV (n = 8). E) Normalized intensity of Nr2f1a staining in ventricles of hearts from control and BIO treated embryos. Control (n = 8); BIO (n = 7). Error bars in graphs indicate s.e.m. * indicate P < 0.05, **** indicate P < 0.0001. (TIF) [file pgen.1011222.s006.tif]

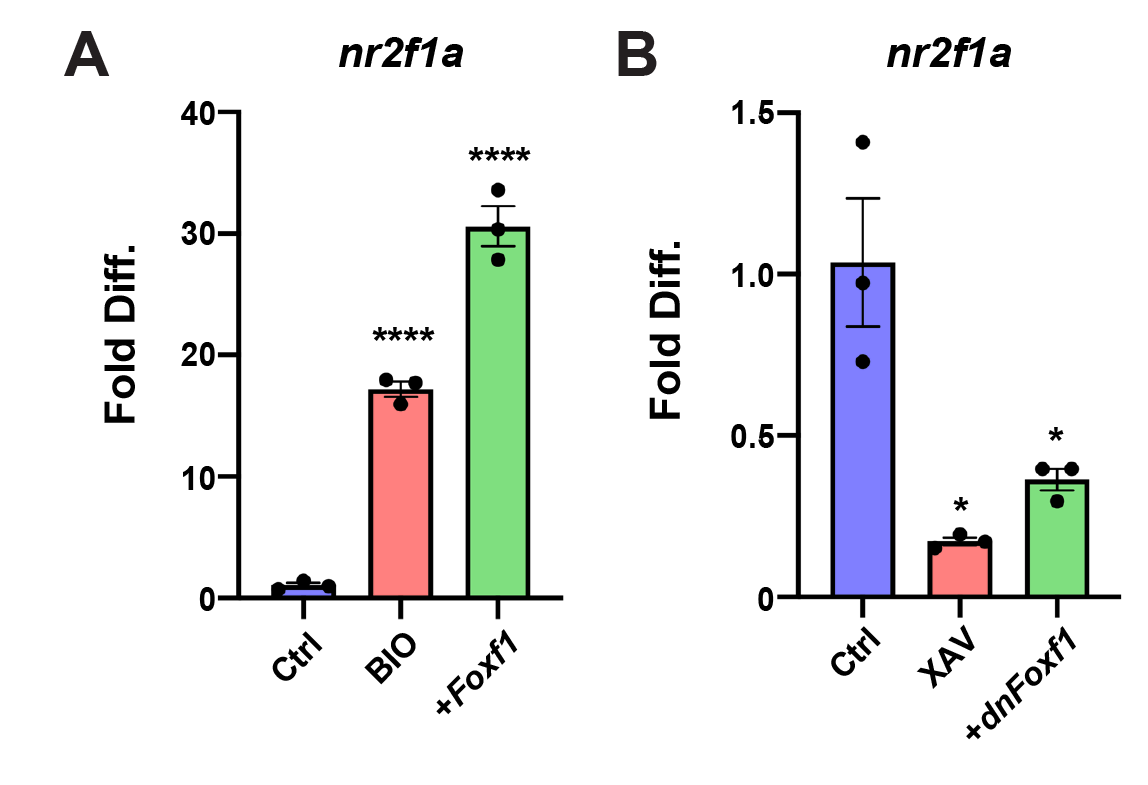

Supplement: S7 Fig — A,B) RT-qPCR for nr2f1a on cDNA from isolated hearts at 24 hpf of embryos treated with BIO and XAV, and injected with Foxf1 mRNA and dnFoxf1 mRNA. Fold difference is relative to β-actin. Control in A,B is the same. Error bars in graphs indicate s.e.m. * indicate P < 0.03, **** indicate P < 0.0001. (TIF) [file pgen.1011222.s007.tif]

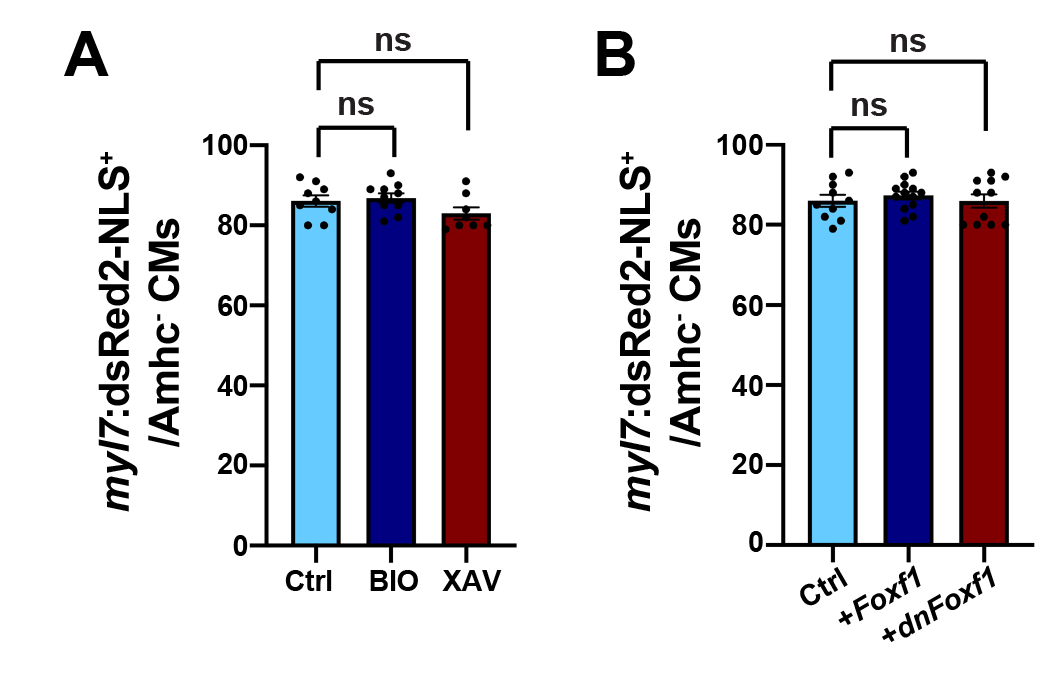

Supplement: S8 Fig — A) The number of VCs (myl7:DsRed2-NLS+/Amhc- cardiomyocytes) within the hearts of control, BIO-, and XAV-treated embryos. Control (n = 9); BIO (n = 10); XAV (n = 8). B) The number of VCs (myl7:DsRed2-NLS+/Amhc- cardiomyocytes) within the hearts of control, Foxf1 mRNA, and dnFoxf1 mRNA injected embryos. Control (n = 10); Foxf1 mRNA (n = 13); dnFoxf1 mRNA (n = 11). Error bars in graphs indicate s.e.m. (TIF) [file pgen.1011222.s008.tif]

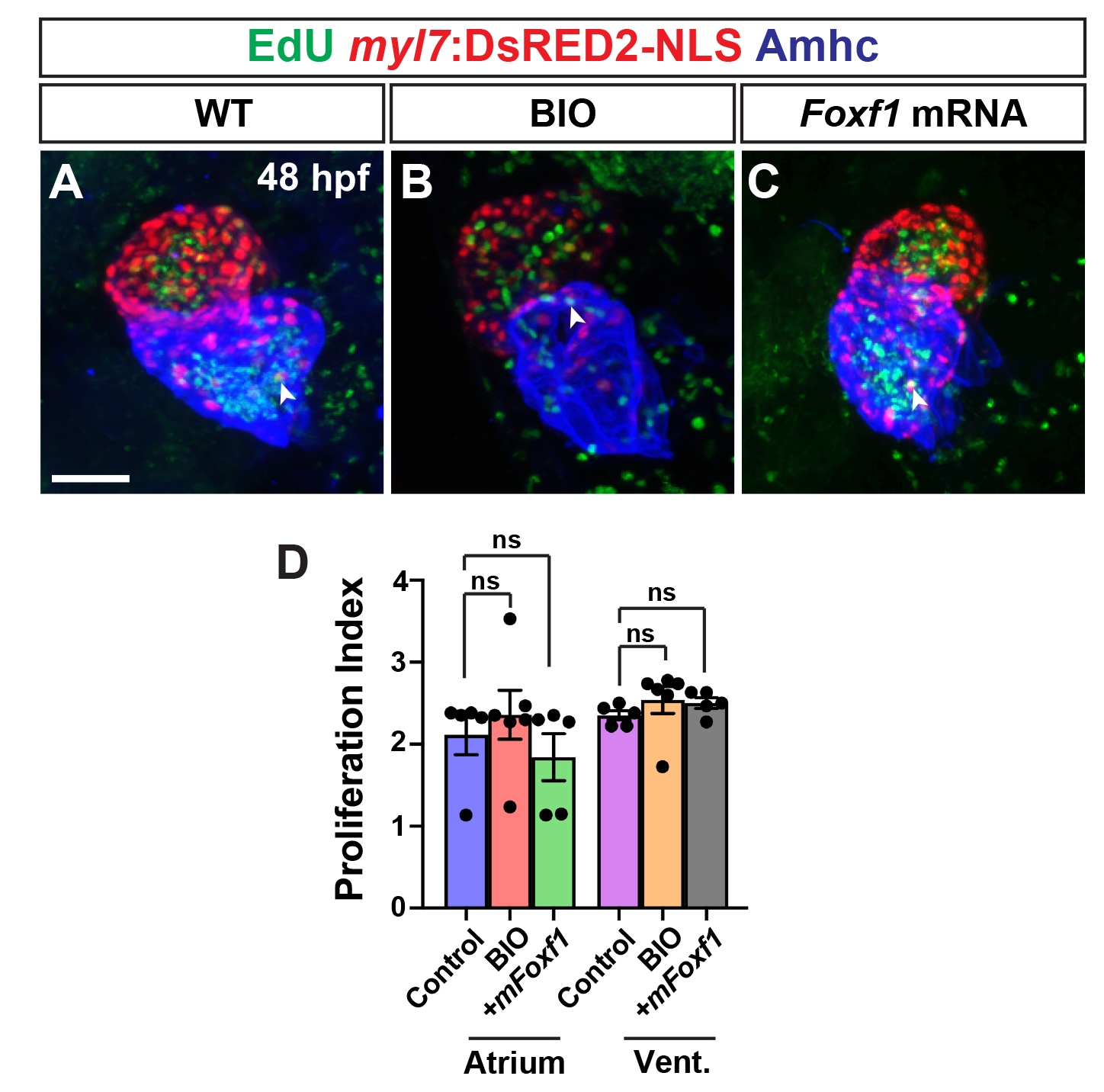

Supplement: S9 Fig — A-C) Confocal images of hearts from control, BIO-treated, and Foxf1 mRNA injected myl7:DsRed2-NLS embryos at 48 hpf that were pulsed with EdU at 24 hpf. EdU (green), myl7:DsRed2-NLS (red), Amhc (blue). Arrows indicate co-labeled EdU+ and DsRed2-NLS+ cardiomyocyte nuclei. Scale bar: 50 μm. D) Proliferation Index of atrial and ventricular cardiomyocytes from Control, BIO treated, and Foxf1 mRNA injected myl7:DsRed2-NLS embryos at 48 hpf that were pulsed with EdU at 24 hpf. Ventricle—Control (n = 5), BIO (n = 6), Foxf1 mRNA (n = 5). Atrium–Control (5), BIO (n = 6), Foxf1 mRNA (n = 5). ns indicates not significance difference relative to controls. Error bars in graphs indicate s.e.m. (TIF) [file pgen.1011222.s009.tif]

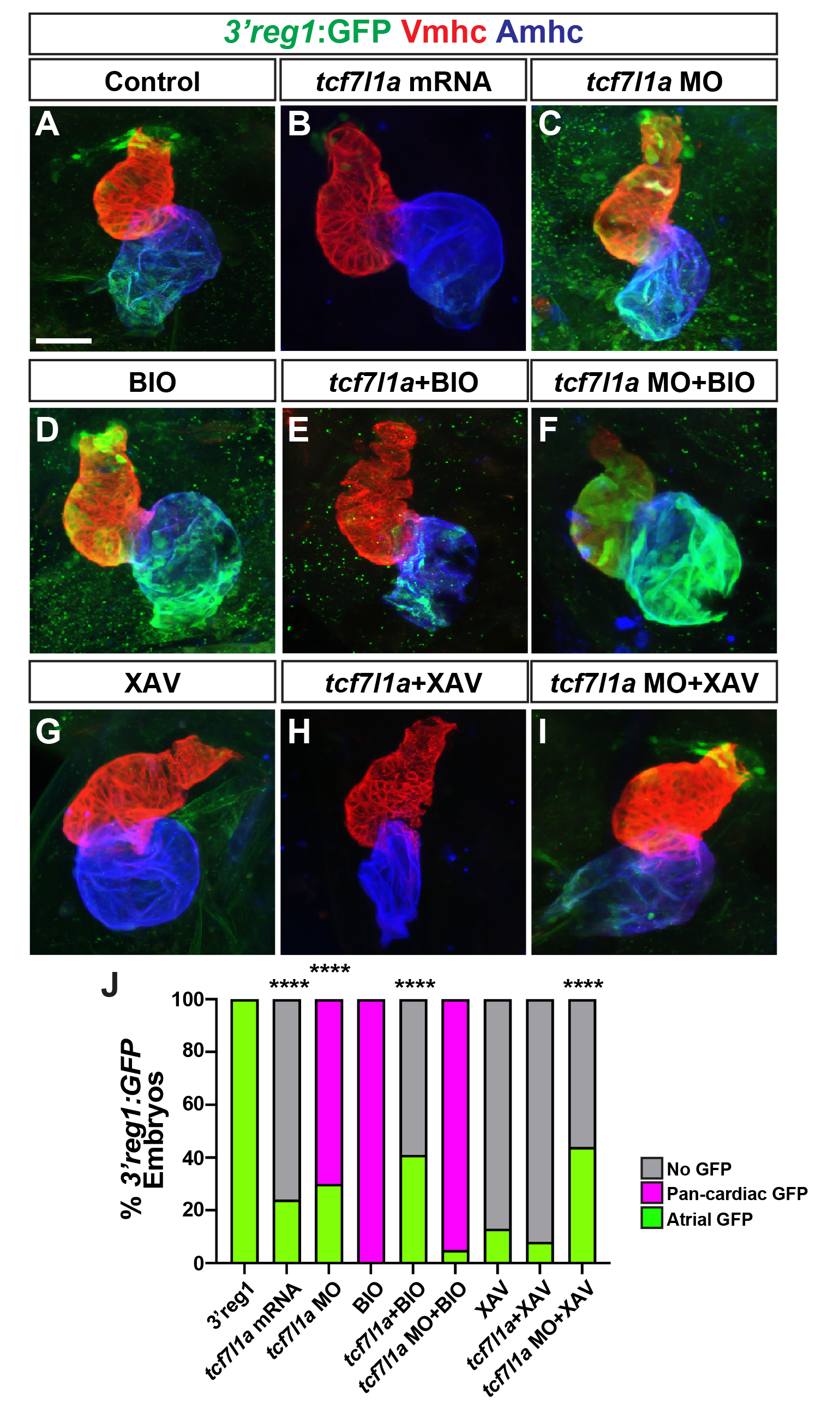

Supplement: S10 Fig — A-I) Confocal images of hearts at 48 hpf from 3’reg1:GFP control, tcf7l1a mRNA injected, tcf7l1a MO injected, BIO-treated, BIO-treated and tcf7l1a mRNA injected, BIO-treated and tcf7l1a MO injected, XAV-treated, XAV-treated and tcf7l1a mRNA injected, XAV-treated and tcf7l1a MO injected embryos. 3’reg1:GFP embryos stained for 3’reg1:GFP (green), Vmhc (red), and Amhc (blue). J) The percentage of 3’reg1:GFP embryos with atrial, pan-cardiac, and lacking expression in their hearts. 3’reg1:GFP control (n = 17), tcf7l1a mRNA injected (n = 20), tcf7l1a MO injected (n = 20), BIO-treated (n = 26), BIO-treated and tcf7l1a mRNA injected (n = 32), BIO-treated and tcf7l1a MO injected (n = 34), XAV-treated (n = 24), XAV-treated and tcf7l1a mRNA injected (n = 24), XAV-treated and tcf7l1a MO injected embryos (n = 30). **** indicate P < 0.0001. (TIF) [file pgen.1011222.s010.tif]

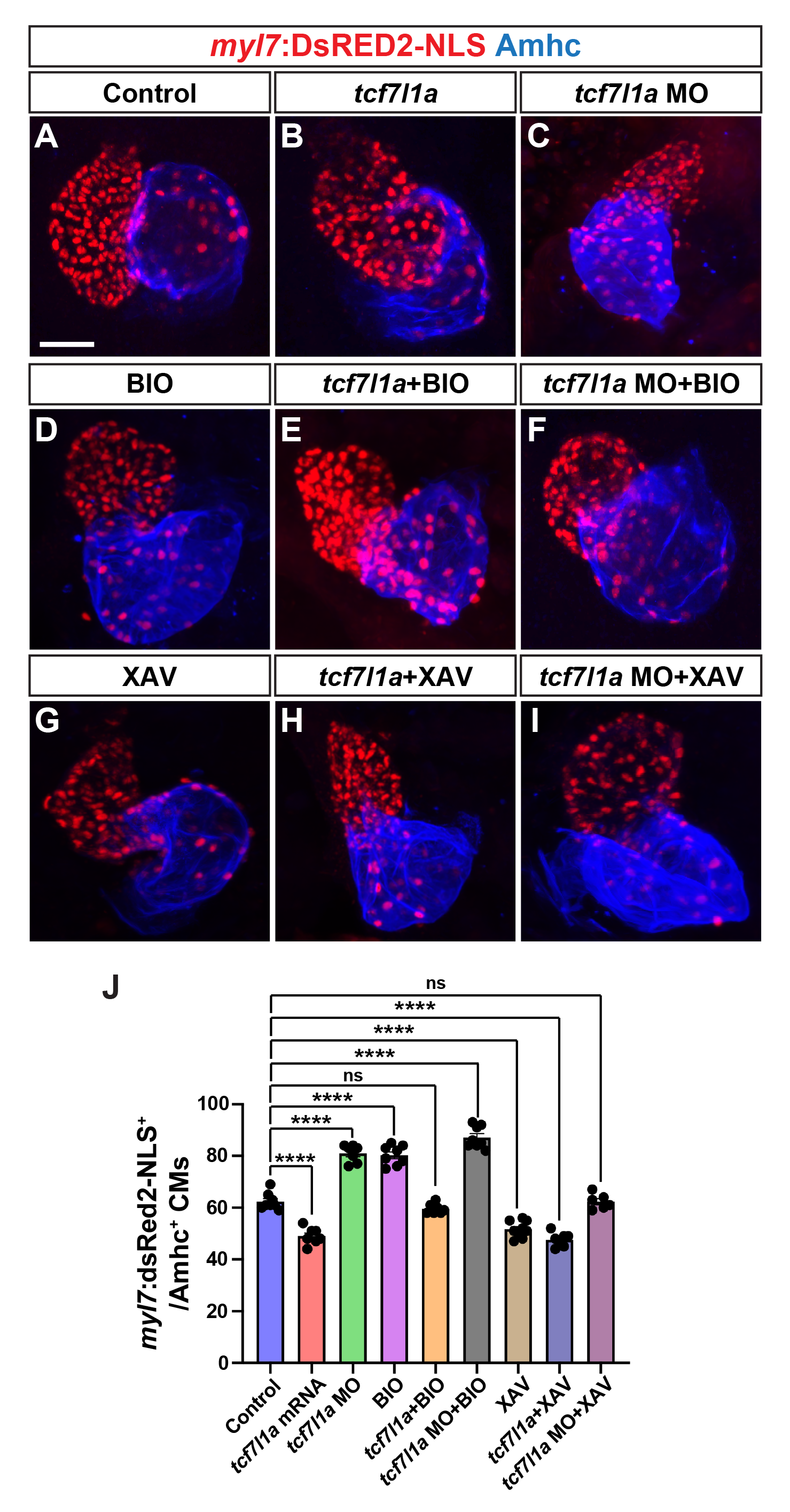

Supplement: S11 Fig — A-I) Confocal images of hearts at 48 hpf from myl7:DsRed2-NLS control, tcf7l1a mRNA injected, tcf7l1a MO injected, BIO-treated, BIO-treated and tcf7l1a mRNA injected, BIO-treated and tcf7l1a MO injected, XAV-treated, XAV-treated and tcf7l1a mRNA injected, XAV-treated and tcf7l1a MO injected embryos. 3’reg1:GFP embryos stained for myl7:DsRed2-NLS (red) and Amhc (blue). J) The number of myl7:DsRed2-NLS+/Amhc+ cardiomyocytes (ACs) within the hearts of Control (n = 8), tcf7l1a mRNA injected (n = 7), tcf7l1a MO injected (n = 7), BIO-treated (n = 8), BIO-treated and tcf7l1a mRNA injected (n = 7), BIO-treated and tcf7l1a MO injected (n = 8), XAV-treated (n = 9), XAV-treated and tcf7l1a mRNA injected (n = 7), XAV-treated and tcf7l1a MO injected myl7:DsRed2-NLS embryos (n = 6). **** indicate P < 0.0001. (TIF) [file pgen.1011222.s011.tif]

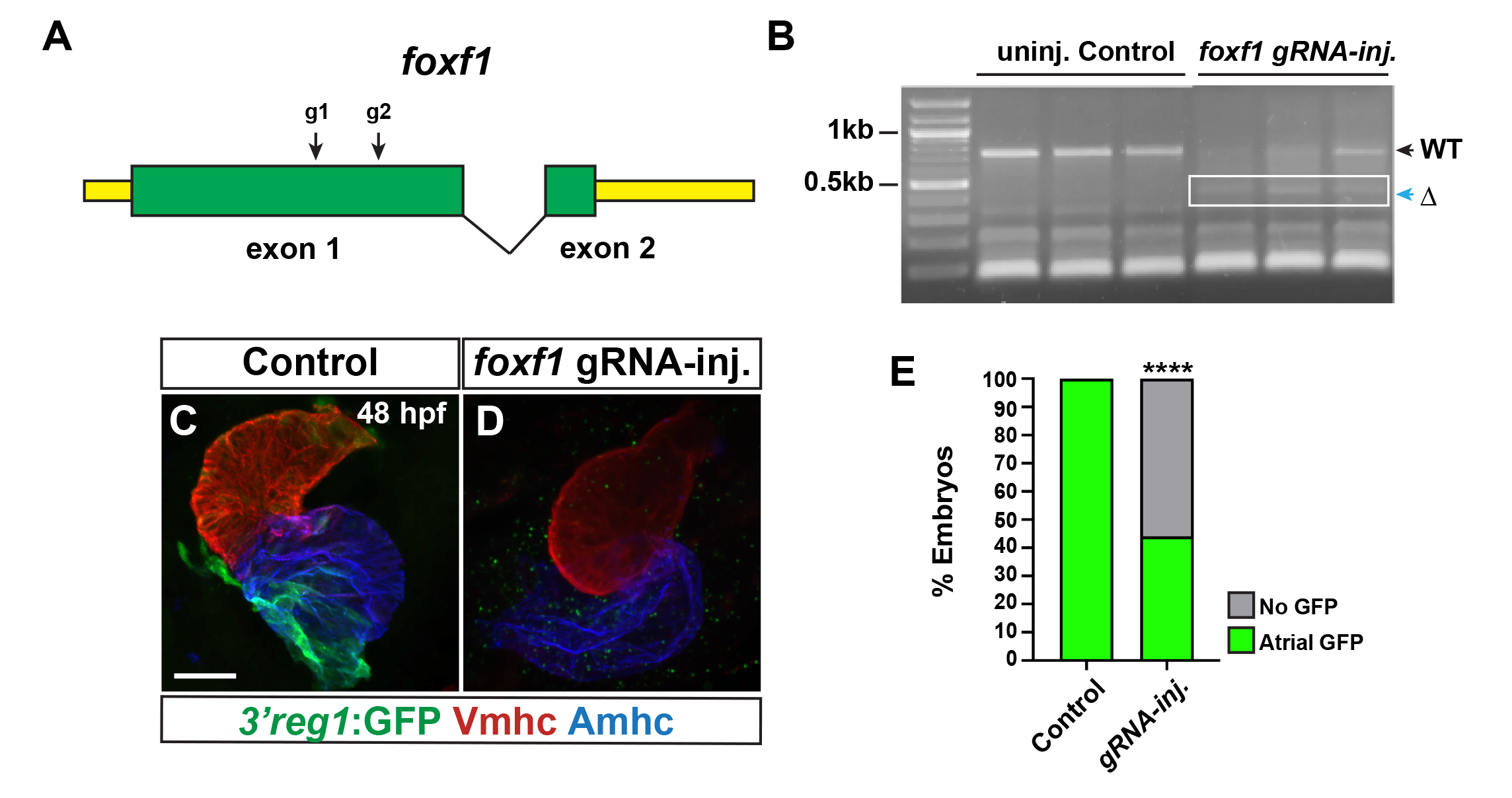

Supplement: S12 Fig — A) Schematic showing the location of the guides (arrow) spaced ~200 bp apart in the first exon of zebrafish foxf1. B) PCR showing the efficacy of guides in creating an ~200 bp deletion and eliminating the WT band for foxf1 within representative injected 3’reg1:GFP embryos. C) Confocal images of hearts from control and foxf1 CRISPR-Cas12 injected transgenic 3’reg1:GFP embryos stained for 3’reg1:GFP (green), Vmhc (red), and Amhc (blue). Scale bars: 50 μm. D) The percentage of control uninjected and foxf1 crispant 3’reg1:GFP embryos with expression in the heart. Control (n = 44); Foxf1 crispant (n = 48). **** indicate P < 0.0001. (TIF) [file pgen.1011222.s012.tif]

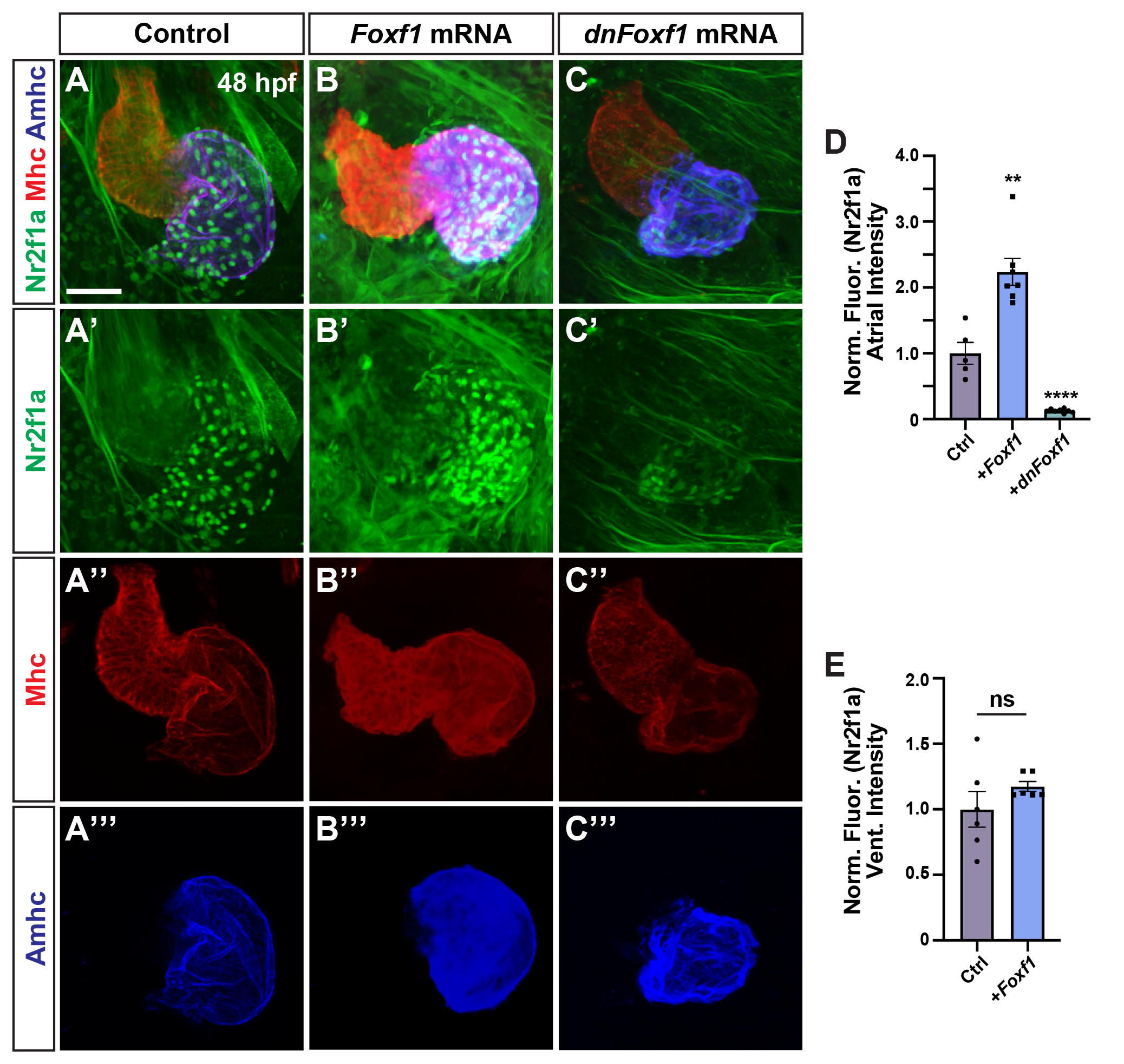

Supplement: S13 Fig — A-C”“) Merged and individual channels of the confocal images of hearts from Fig 6F–6H. Hearts from transgenic 3’reg1:GFP embryos stained for (A’-C’) Nr2f1a (green), (A”-C”) Mhc (pan-cardiac–red) (magenta), and (A”’-C”’) Amhc (ACs–blue). Scale bar: 50 μm. D) Normalized intensity of Nr2f1a staining in atria of hearts from control, Foxf1 mRNA, and dnFoxf1 mRNA injected embryos. Control (n = 5); Foxf1 mRNA (n = 7); dnFoxf1 mRNA (n = 8). E) Normalized intensity of Nr2f1a staining in ventricles of hearts from control and Foxf1 mRNA injected embryos. Control (n = 6); Foxf1 mRNA-injected (n = 6). Error bars in graphs indicate s.e.m. * indicate P < 0.05, **** indicate P < 0.0001. (TIF) [file pgen.1011222.s013.tif]

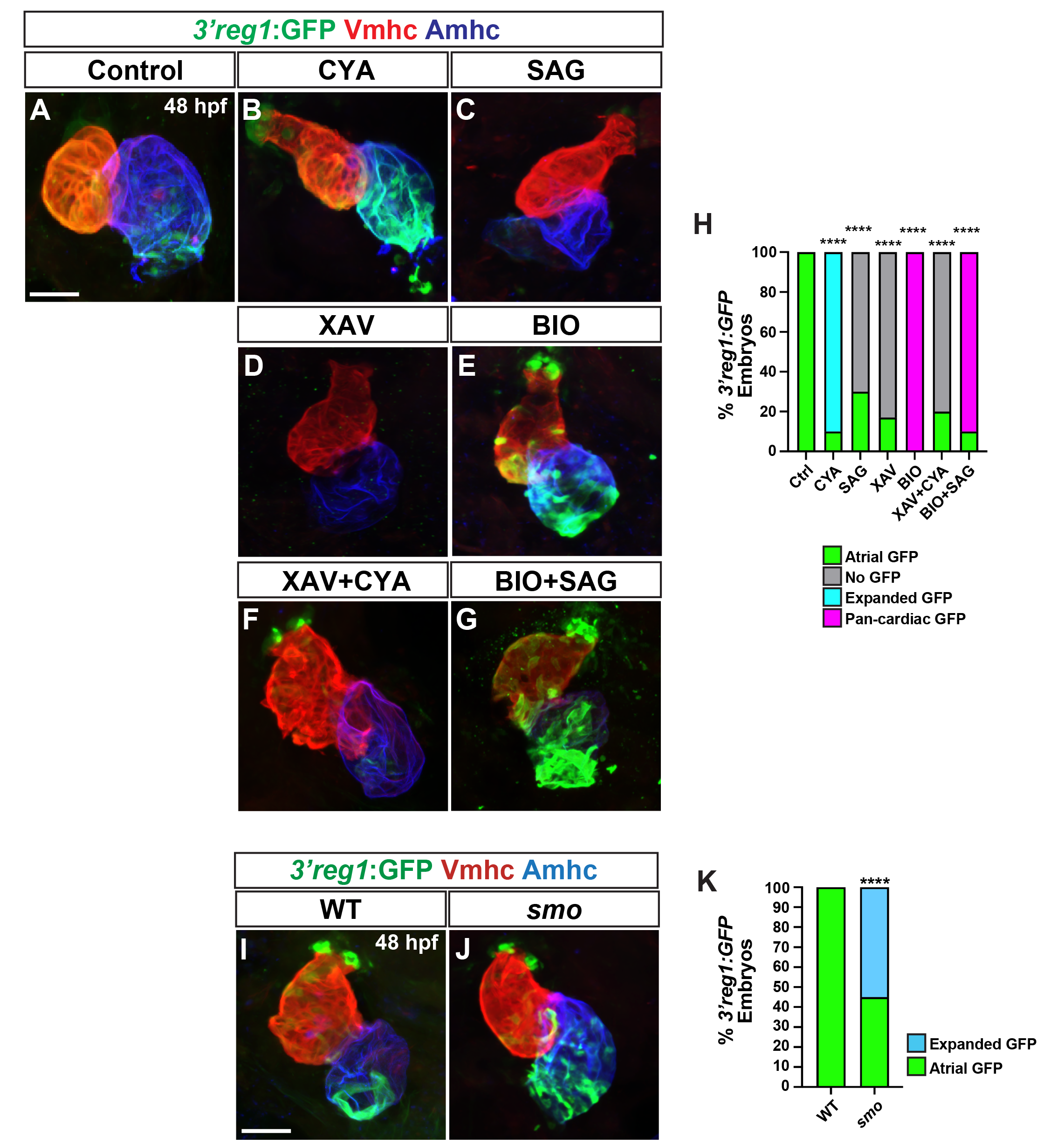

Supplement: S14 Fig — A-G) Confocal images of hearts from untreated control, CYA-treated, SAG-treated, XAV-treated, BIO-treated, XAV+CYA-treated, and BIO+SAG-treated transgenic 3’reg1:GFP embryos stained for 3’reg1:GFP (green), Vmhc (red), and Amhc (blue). H) The percentage of control, CYA-treated, SAG-treated, XAV-treated, BIO-treated, XAV+CYA-treated, and BIO+SAG-treated 3’reg1:GFP embryos that had expression in the atria, inhibited expression, expanded expression within the atria, and pan-cardiac expression. Control (n = 31); CYA (n = 44); SAG (n = 98). XAV-treated (n = 48), BIO-treated (n = 28), XAV+CYA-treated (n = 99), and BIO+SAG-treated (n = 109). I,J) Confocal images of hearts from WT sibling and smo mutant 3’reg1:GFP embryos stained for 3’reg1:GFP (green), Vmhc (red), and Amhc (blue). K) The percentage of WT and smo 3’reg1:GFP embryos that had expanded reporter expression within their atria. Control (n = 24); smo (n = 51). Scale bars: 50 μm. Error bars in graph indicate s.e.m. **** indicate P < 0.0001. ns indicates not a statistically significant difference. (TIF) [file pgen.1011222.s014.tif]

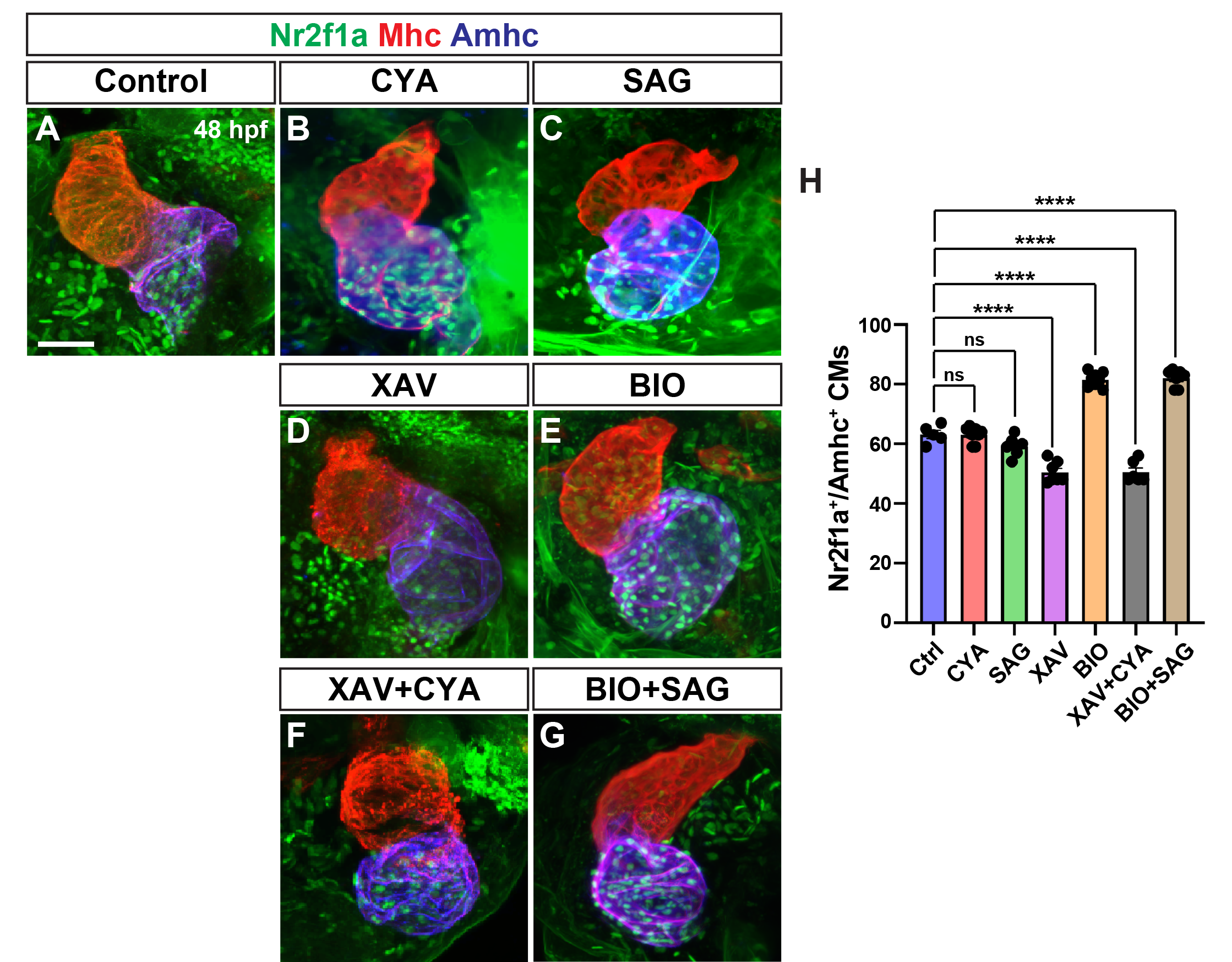

Supplement: S15 Fig — A-G) Confocal images of hearts from untreated control, CYA-treated, SAG-treated, XAV-treated, BIO-treated, XAV+CYA-treated, and BIO+SAG-treated embryos stained for Nr2f1a (green), Mhc (red), and Amhc (blue). Scale bar: 50 μm. H) The number of Nr2f1a+/Amhc+ cardiomyocytes in the hearts of untreated control, CYA-treated, SAG-treated, XAV-treated, BIO-treated, XAV+CYA-treated, and BIO+SAG-treated embryos. Control (n = 5), CYA (n = 8), SAG (n = 7), XAV-treated (n = 7), BIO-treated (n = 10), XAV+CYA-treated (n = 6), and BIO+SAG-treated (n = 7). Error bars in graph indicate s.e.m. **** indicate P < 0.0001. ns indicates not a statistically significant difference. (TIF) [file pgen.1011222.s015.tif]

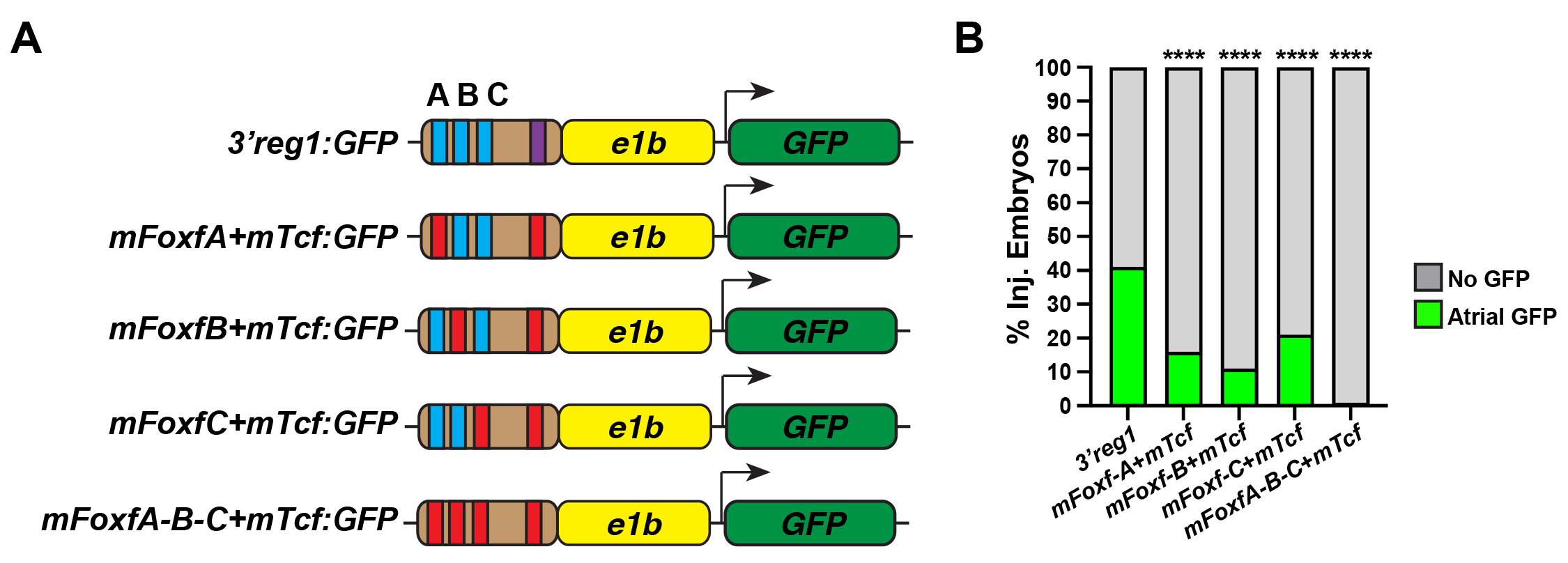

Supplement: S16 Fig — A) Schematics of 3’reg1:GFP reporter constructs with WT, mutated Foxf-A+mutated Tcf sites, mutated Foxf-B+mutated Tcf sites, mutated Foxf-C+mutated Tcf sites, and mutated of all 3 Fox sites (Foxf-A-B-C) + mutated Tcf sites. WT Foxf sites (blue). WT Tcf site (purple). Mutated sites (red). Mutations were made as in Figs 2 and 5. B) The percentage of transient transgenic embryos with reporter expression in the atria. 3’reg1 (n = 34); mFoxf-A+Tcf (n = 80); mFoxf-B+Tcf (n = 50); mFoxf-C+Tcf (n = 45); mFoxf-A-B-C+Tcf (n = 28). **** indicate P < 0.0001. (TIF) [file pgen.1011222.s016.tif]

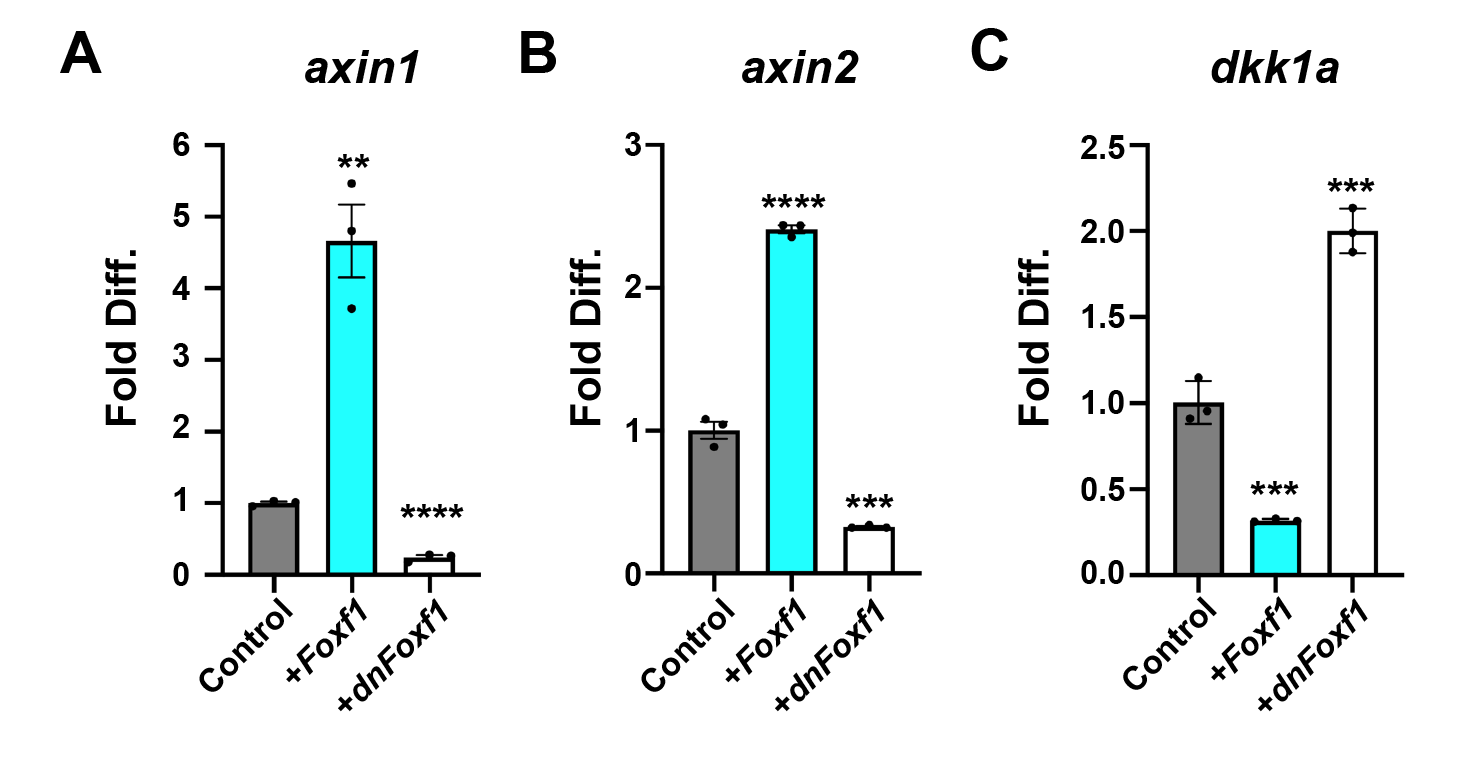

Supplement: S17 Fig — A,B) RT-qPCR for axin1, axin2, and dkk1a in isolated hearts from 24 hpf embryos injected with Foxf1 mRNA and dnFoxf1 mRNA. Fold difference is relative to β-actin. Error bars in graphs indicate s.e.m. ** indicates P < 0.002.*** indicates P < 0.001, **** indicates P < 0.0001. (TIF) [file pgen.1011222.s017.tif]

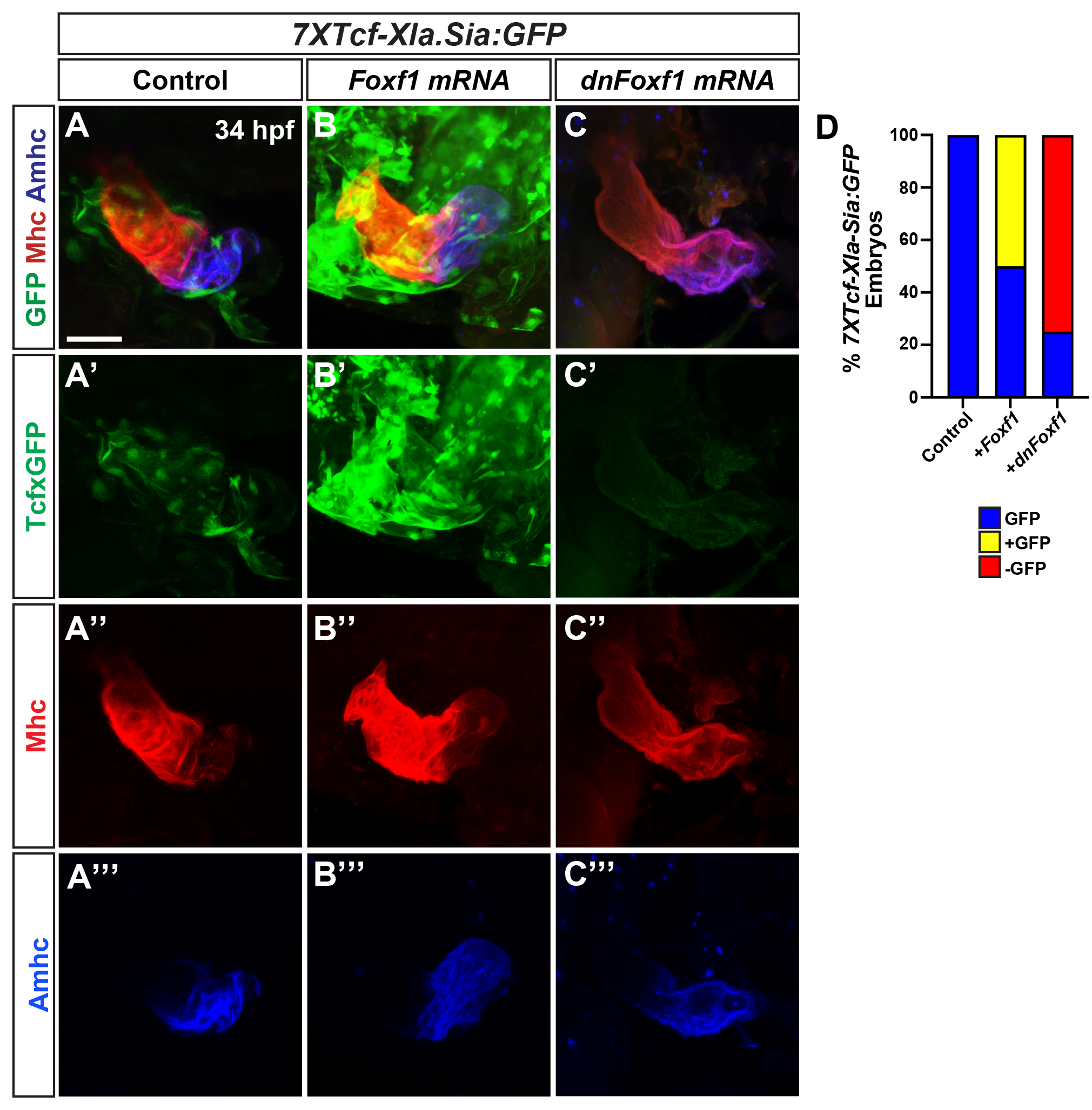

Supplement: S18 Fig — A-C”“) Merged and individual channels of the confocal images of hearts from control, Foxf1 mRNA injected, and dnFoxf1 mRNA injected 7xTcf.Xla-Sia:GFP embryos at 34 hpf. (A’-C’) 7xTcf.Xla-Sia:GFP (green), (A”-C”) Mhc (red), and (A”’-C”’) Amhc (blue). Scale bar: 50 μm. D) Percentage of Control, Foxf1 mRNA injected, and dnFoxf1 mRNA injected 7xTcf.Xla-Sia:GFP embryos at 34 hpf with normal, increased, or decreased expression near and in the hearts. Control (n = 35), Foxf1 mRNA (n = 97), dnFoxf1 mRNA (n = 106). (TIF) [file pgen.1011222.s018.tif]

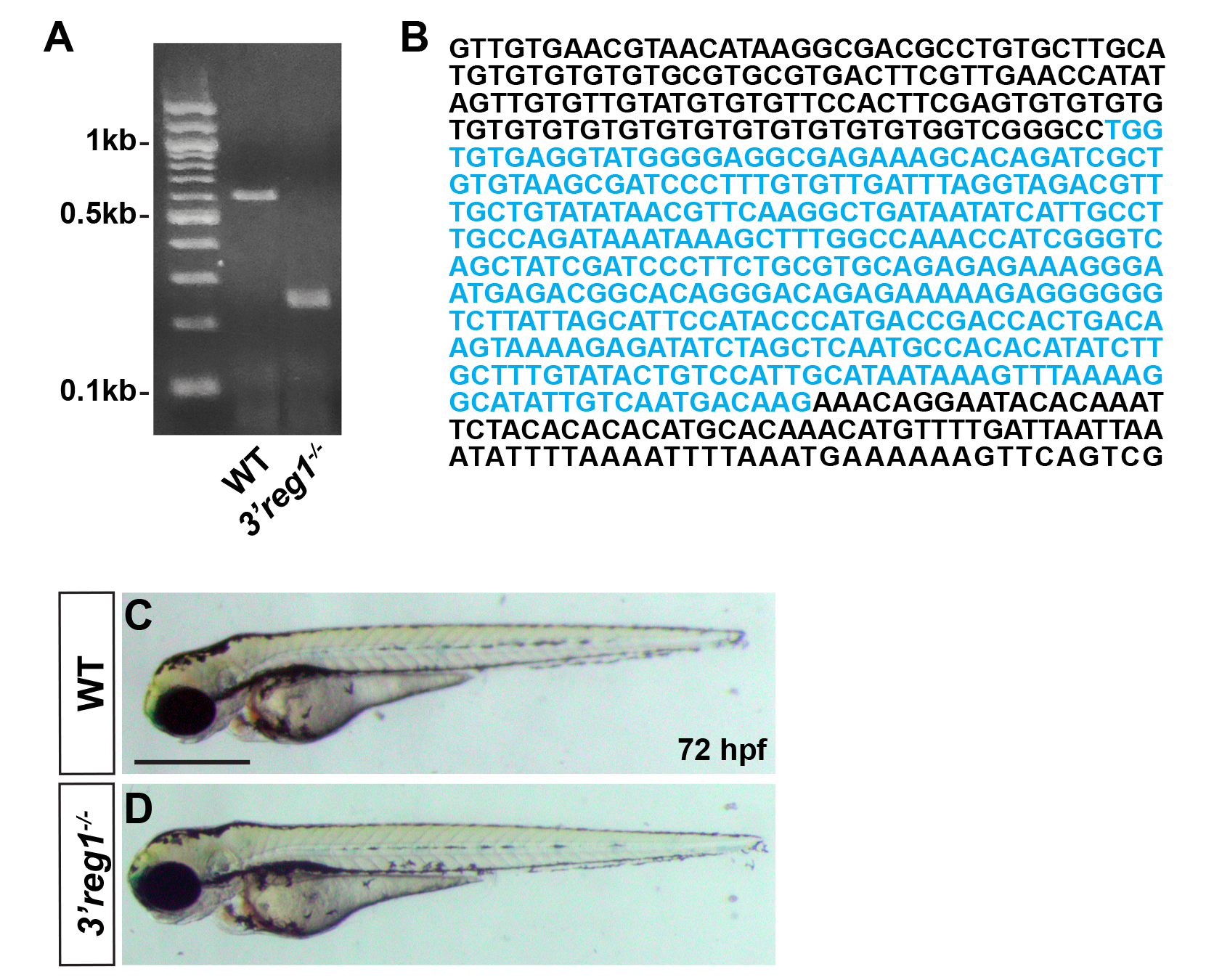

Supplement: S19 Fig — A) PCR from WT and 3’reg1 embryos. The WT 3’reg1 PCR product is 594 bp. The PCR product for the 3’reg1 deletion is 354 bp. B) 3’reg1 sequence showing the deleted sequence (blue). C,D) Representative WT and 3’reg1-/- embryos at 72 hpf. Lateral views with anterior leftward and dorsal upward. 3’reg1-/- embryos do not have an overt phenotype. Scale bar: 500 μm. (TIF) [file pgen.1011222.s019.tif]

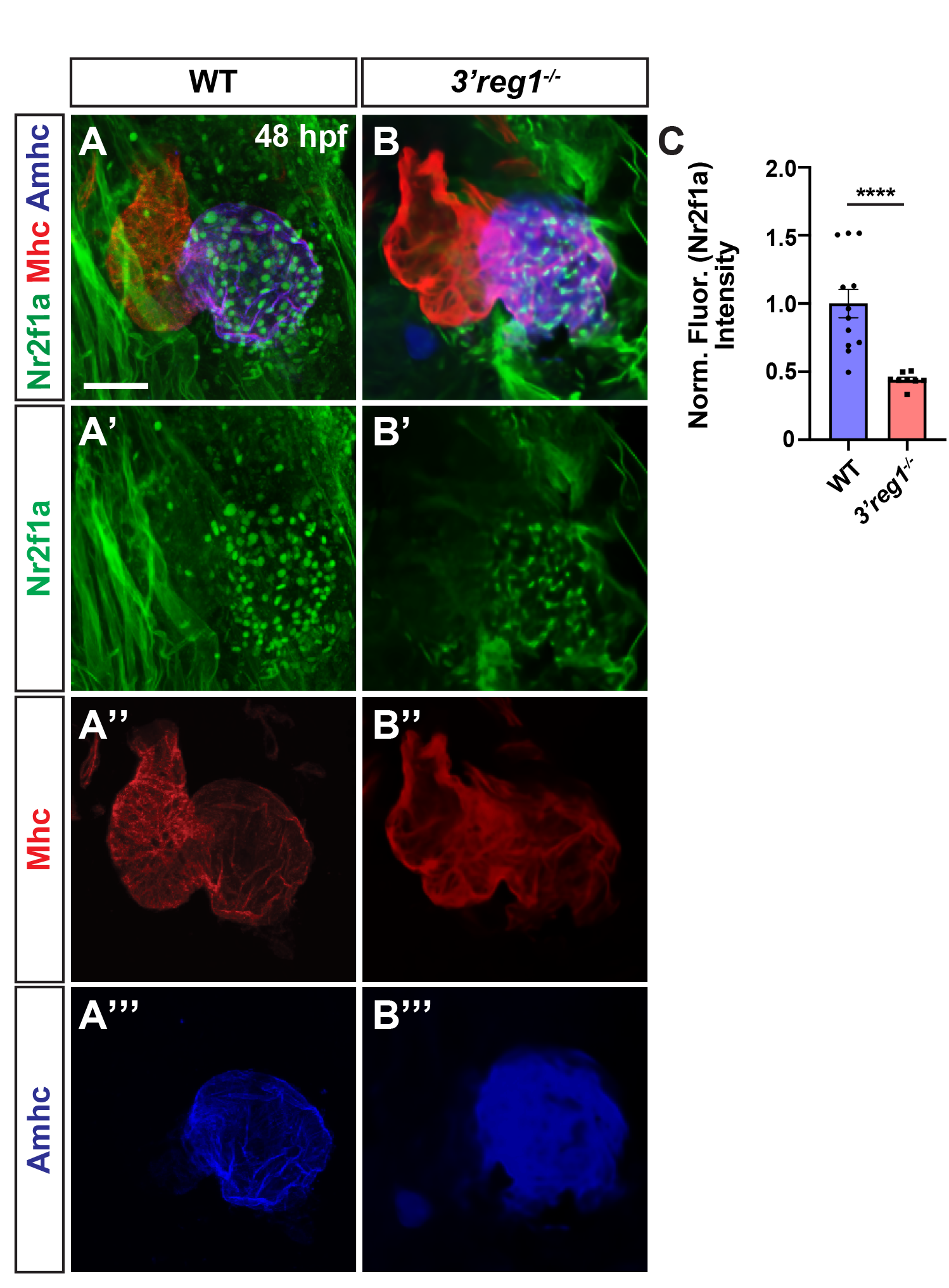

Supplement: S20 Fig — A-B”’) Merged and individual channels of the confocal images of hearts from 3’reg1-/- embryos. (A’,B’) Nr2f1a (green), (A”,B”) Mhc (red), (A”’,B”’), and Amhc (blue). Scale bar: 50 μm. C) Normalized intensity of Nr2f1a staining in atria of hearts from WT sibling and 3’reg1-/- embryos. WT sibling (n = 12); 3’reg1-/- (n = 8). Error bars in graphs indicate s.e.m. **** indicate P < 0.0001. (TIF) [file pgen.1011222.s020.tif]

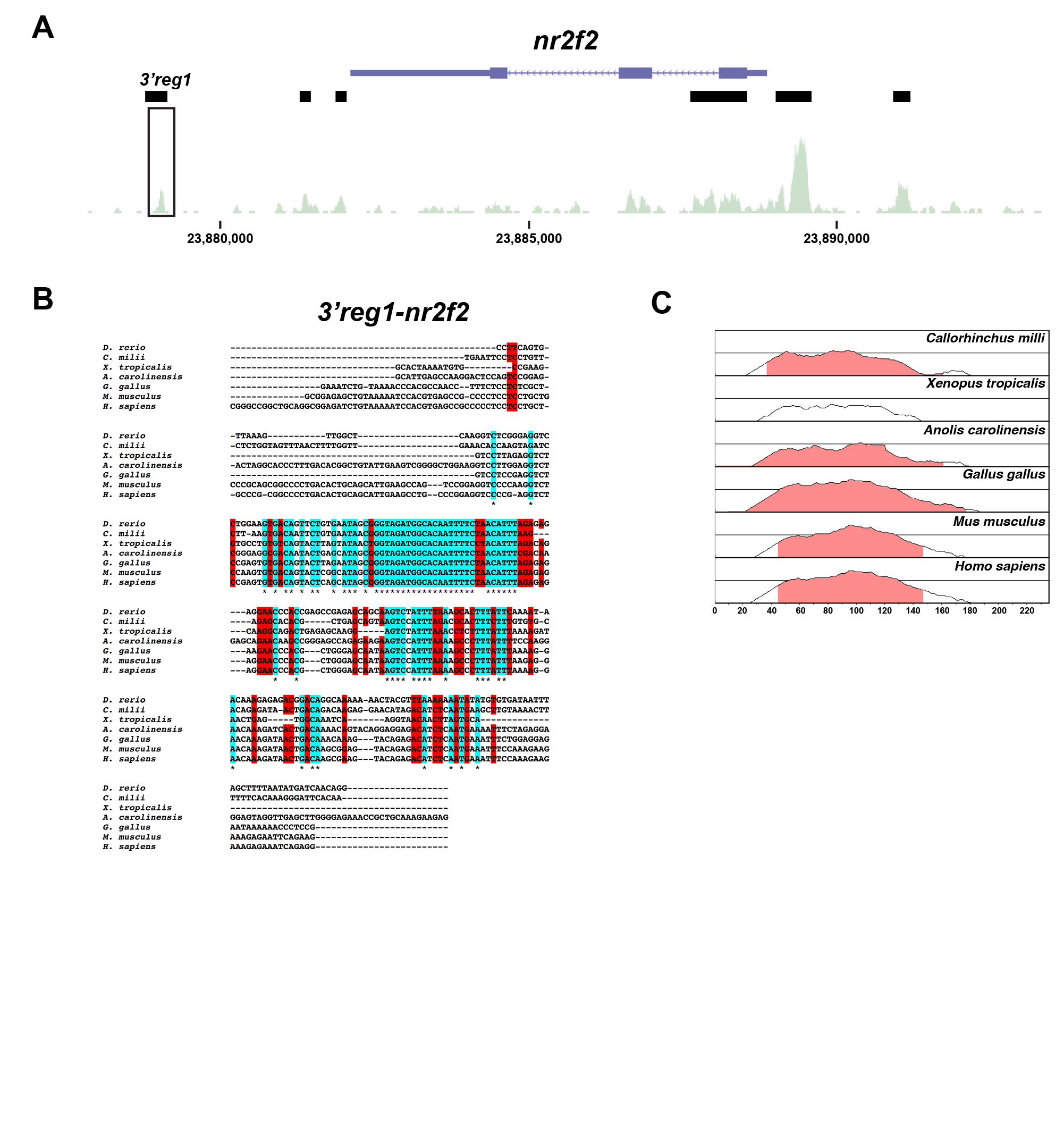

Supplement: S21 Fig — A) Schematic of ATAC-seq data in ACs showing the localization of the putative 3’reg1-nr2f2 enhancer relative to the nr2f2 locus. Image of tracks generated in UCSC genome browser (https://genome.ucsc.edu). B) Clustal alignment of 3’reg1-nr2f2 from Danio rerio (zebrafish), Callorhinchus milli (Australian ghostshark), Xenopus tropicalis (Tropical clawed frog), Anolis carolinensis (Green Anole), Gallus gallus (chicken), Mus musculus (House mouse), and Homo sapiens (human). Turquoise indicates completely conserved nucleotides. Red indicates partially conserved nucleotides. C) VISTA plot showing conservation of the zebrafish 3’reg1-nr2f2 enhancer with regions in Callorhinchus milli (Australian ghostshark), Xenopus tropicalis (Tropicalis clawed frog), Anolis carolinensis (green Anole), Gallus gallus (chicken), Mus musculus (House mouse), and Homo sapiens (human). Pink indicates >50% conservation of regulatory regions with zebrafish 3’reg1. Median lines in individual VISTA plots indicate 75% conservation. (TIF) [file pgen.1011222.s021.tif]
