# Supplementary material for: A Foxf1-Wnt-Nr2f1 cascade promotes atrial cardiomyocyte differentiation in zebrafish
Source: PLoS Genet. 2024 Nov 4;20(11):e1011222. doi: 10.1371/journal.pgen.1011222 (PMC11563408; doi:10.1371/journal.pgen.1011222)
Supplement: Attachment — Submitted filename: Response to Reviewers_PGENETICS-D-24-00293.docx [file pgen.1011222.s028.docx]

**Response to Reviewer Comments – Manuscript PGENETICS-D-24-00293**

We appreciate the thoughtful critiques of our original manuscript. There seemed to be consensus that there were strengths to our analysis of the *nr2f1a* *3’reg1* enhancer and the regulatory mechanism of atrial differentiation it suggested. In particular, Reviewer #2’s comments highlighted that it was a “*comprehensive study*” with “*high quality data and clear writing*.” Reviewer #3 echoed these assessments stating the study would be of “*high interest*” and that “*the experiments are well performed and documented*.” While the overall assessments from the reviewers were positive, the reviewers felt there were weaknesses and recommended a number of experiments that could help to strengthen the manuscript. One of the primary weaknesses mentioned was the lack of quantification of expression within the heart for the GFP reporter and Nr2f1a. In our revised manuscript, we have tried to directly address all the concerns of the reviewers. In particular, the changes made in response to their critiques of the original manuscript have allowed us to clarify ambiguity regarding the localization of the reporter within the heart relative to Nr2f1a and the levels of the reporter and Nr2f1a expression following manipulation of Tcf7l1a, Wnt signaling, and Foxf1. We have provided a point-by-point response to each of the Reviewers’ comments. Responses are indicated below the original reviewer’s comments in blue. Changes made to the text in the revised manuscript in direct response to the reviewers’ comments are indicated in gray.

-----------

**Reviewer #1**: In the manuscript authored by Coppola et al., the researchers identified a conserved enhancer 3’ to the nr2f1a locus, named 3’reg1-nr2f1a (3’reg1), that was able to drive reporter gene expression within ACs of stable transgenic lines. The authors also identified a Lef/Tcf binding site and multiple Foxf binding sites within the enhancer, which, when mutated, could lead to ectopic or diminished reporter gene expression in the developing heart. Further gain- and loss-of-function experiments manipulating Wnt signaling and Foxf1 activity supported the observations made by mutating the respective DNA binding motifs. The authors suggest that 3’reg1 acts as the enhancer element promoting Nr2f1a expression in ACs; however, embryos with a homozygous deletion of the endogenous 3’reg1 fragment showed no overt defects and did not exhibit affected Nr2f1a expression in the heart. Therefore, the overall statement that “our data support that downstream members of a conserved regulatory network involving Wnt signaling and Foxf1 function on a nr2f1a enhancer to promote AC differentiation in the zebrafish heart” is not sufficiently supported by data presented in the manuscript.

We did not respond to the brief critique from Reviewer #1 because specific points for us to address were not provided.

**Reviewer #2**: In this manuscript, Coppola et al investigated the regulation of Nr2f1a transcription factor in the differentiation of vertebrate atrial cardiomyocytes (ACs). They identified an enhancer, named 3’reg1, located 3’ to the nr2f1a locus, which promotes expression within ACs. This enhancer contains binding sites for Lef/Tcf and Foxf transcription factors. Mutations in these sites affect the enhancer's activity. The authors proposed a model where Wnt signaling, downstream of Foxf1, relieves Tcf7l1a-mediated repression of the enhancer, promoting AC differentiation. Hh signaling, although known for its role in venous SHF differentiation, only modestly affects the enhancer's activity and does not impact AC differentiation. The authors found that Nr2f1a functions downstream of Wnt signaling and Foxf1. CRISPR-mediated deletion of the enhancer abolishes the ability of Wnt signaling and Foxf1 to enhance AC differentiation, suggesting its essential role in the process. Overall, this study outlines a new regulatory network involving Wnt signaling and Foxf1 that promotes AC differentiation through the regulation of zebrafish nr2f1a expression via a conserved enhancer. It's a comprehensive study employing both gain- and loss-of-function assays, with high data quality and clear writing. However, several concerns need addressing.

1. The extent to which the 3’reg1:GFP reporter reflects endogenous nr2f1a expression remains unclear. Are they expressed in the same cells with similar dynamics during heart development? How many stable lines were analyzed?

The *3’reg1:GFP* reporter does not fully recapitulate all of Nr2f1a’s endogenous expression. We apologize this was not made clear in the original manuscript. The 3’reg1 reporter recapitulates some of *nr2f1a* expression within atrial cardiomyocytes, which is not unusual for different cis-regulatory elements. Endogenous *nr2f1a* starts in the anterior lateral plate mesoderm shortly after the tailbud stage. It later becomes expressed in differentiating atrial progenitors and cardiomyocytes as the tube elongates (Duong et al, 2018). As we have reporter previously, Nr2f1a appears to be expressed in all atrial cardiomyocytes. As shown in Figure 1, we find that the *3’reg1:GFP* reporter begins expression around 24. Its expression appears to be in the atrial cardiomyocytes, but have higher levels toward the venous pole, as we state in the manuscript. While there is a little variability to the transgenic reporter, it is expressed in most atrial cardiomyocytes overlapping with Nr2f1a. In some embryos, there is some low level, sporadic expression in a few ventricular cardiomyocytes, which is also observed with the *amhc* reporter (Zhang et al, 2013, Foglia et al, 2016).

We have revised the text (lines 152-154) and revised Fig. 1 to include co-staining of the transgenic *3’reg1:GFP* reporter and endogenous Nr2f1a. We have also included the different channels from the images in a new S4 Fig. of the revised manuscript to make the comparison between the *3’reg1:GFP* reporter and Nr2f1a clear.

For all stable transgenic lines, at least two independent lines from different founders were identified. Both *3’reg1:GFP* transgenic lines had similar expression in the atria. Therefore, we proceeded to preform analysis with one transgenic line, which was used in the manuscript for all experiments. Unfortunately, we did not maintain the second line due to space constraints.

2. Figure 2F and Figure 4C show that mutating Tcf and Foxf binding sites did not fully abolish atrial-specific GFP expression, suggesting that these binding sites may not be essential for the atrial activity of the enhancer. Please clarify. Generation of stable transgenic lines may be necessary for sorting this out. Also, analysis of double mutants of the Tcf and Foxf binding sites may be informative to dissect any additive and/or redundant functions of these binding sites.

We have analyzed loss of multiple Fox sites and the Tcf site together. We have found the mutation of any of the Foxf sites individually along with mutating the Tcf site gives a result similar to mutation of the Foxf sites alone, reduced expression in the atrium. Mutation all three Foxf sites along with mutation of the Tcf site results in a complete loss of expression from the reporter in transient transgenic embryos. Therefore, this data suggests that at least in transient transgenic embryos that the Foxf sites are necessary for activation of the reporter. While this may seem a bit contradictory to the epistasis in the stable transgenic line, one interpretation of these results would still be that Foxf signaling functions upstream of Wnt signaling and is also needed to activate the reporter/endogenous enhancer. We now mention this experiment in the Results (lines 313-321), have included this as a S16 Fig., and discuss the results in the Discussion (lines 425-432) in the revised manuscript.

We agree that creating stable transgenic lines for all the analysis would have been ideal. Unfortunately, it was not practical for us to create stable transgenic lines for the high number of different constructs needed to be analyzed due to time and space constraints. Additionally, most of the transient transgenic analysis was corroborated by our analysis of the stable lines and using transient transgenic has been an acceptable way of analyzing constructs in the field for similar analysis.

3. The authors claimed that Wnt signaling activates the transgenic 3’reg1:GFP reporter and promote a surplus of differentiated ACs, likely through derepression of Tcf7l1a. However, rescue experiments using tcf7l1a under manipulated wnt conditions are needed.

We have performed manipulation of Tcf7l1a expression while manipulating Wnt conditions. Overall, the data support the hypothesis that Tcf7l1a functions downstream of Wnt signaling. However, we recognize that depleting Tcf7l1a while inhibiting Wnt with XAV does not completely give the predicted result that the effects should resemble Tcf7l1a depletion. Instead, there is an intermediate effect with respect to restoration of the reporter and the quantification of atrial cardiomyocytes. This intermediate response could reflect that the temporal inhibition of Wnt signaling at the 20 somite stage coupled with mosaic depletion of Tcf7l1a at earlier stages. Nevertheless, overall these results support that Tcf7l1a functions downstream of Wnt signaling as a repressor. We have provided these data in the results (lines 242-256) and new S10 and S11 Figs.

4. Lines 266-268, “We found that simultaneously inhibiting or activating Wnt and Hh signaling on decreased and increased expression of the 3’reg1:GFP reporter within the heart, similar to inhibiting or activation of Wnt signaling alone (S7L-O Fig.).” Control groups for inhibiting or activating Wnt signaling alone are lacking in the experiments. Comparisons with the results from other experiments may not be accurate.

We did initially perform the experiments with all the different conditions. However, we omitted the BIO and XAV groups to conserve space in the figure as BIO and XAV experiments were presented in other figures. The analysis of BIO and XAV in this experiment is consistent with what was found in the other experiments. We have added back the BIO and XAV controls to what is now S14 Fig. of the revised manuscript.

5. Lines 268-270, “Therefore, our data suggest that in contrast to mammalian venous pole differentiation Hh signaling is likely not affecting AC differentiation at these stages of cardiogenesis nor is it promoting Nr2f expression in ACs upstream of Wnt or Foxf1 in zebrafish embryos.” The experiments referred here in Fig. S7L-O were performed on the 3’reg1:GFP reporter, not endogenous nr2f1a expression, which should be tested to support claims about Hh signaling.

We have performed the epistasis of HH and Wnt signaling and examined endogenous Nr2f1a expression. Similar to the experiments on the reporter, we found that manipulation of HH signaling alone at these stages did not affect the number of Nr2f1a+ cardiomyocytes nor did it affect the number of Nr2f1a+ cardiomyocytes when manipulating Wnt signaling. These experiments are included in the results (lines 301-307) and S15 Fig of the revised manuscript.

6. Line 177, the negative data should be shown.

We have provided the quantification for the negative data for these experiments. As the expression in the hearts did not look different than the *3’reg1* reporter, it seemed uninformative to add images. We have added the quantification data to S5 Fig. of the revised manuscript.

7. Line 185, Please correct this sentence “we found that either deletion or targeted mutation of the Lef/Tcf site both resulted in…”.

Thank you. This has been corrected.

**Reviewer #3**: The manuscript by Coppola et al describes identification of an enhancer that regulates nr2f1a expression during zebrafish atrial cardiomyocyte differentiation. Authors demonstrate that the conserved 3’ enhancer restricts GFP reporter expression to atrial cardiomyocytes. They show that the enhancer is regulated by foxf1 and wnt signaling, and demonstrate their roles in restricting the number of atrial cardiomyocytes. Altogether, the work establishes the regulatory network that involves wnt and foxf1 signaling that function through nr2f1a to promote differentiation of atrial cardiomyocytes in zebrafish.
Overall the results are novel and significant, and they provide mechanistic insight into pathways involved in cardiac differentiation. These findings will be of high interest to many researchers working on cardiac specification and regeneration. The experiments are well performed and documented. Major weaknesses are the lack of quantification of expression intensities in different treatments and the fact that that different experimental manipulations and treatments appear to have different effects on the reg1:GFP enhancer and endogenous nr2f1a expression (see Point #3). It is suggested that the authors address the following points.

1. It would be helpful to show GFP channel separately in Fig. 1F-H. It is not clear if GFP is completely absent from the ventricle, or if it is expressed at a low level.

Yes, in some embryos there is sporadic, low-level expression of GFP in a few ventricular cardiomyocytes. Interestingly, this was also observed for *amhc*/*myh6* transgenic reporters (Zhang et al, 2013, Foglia et al, 2016). This reporter expression could reflect maintained expression from cardiomyocytes that express both atrial and ventricular markers within the early heart tube (Martin, et al, 2023) or some variability of the transgenic line. Importantly, the majority of the *3’reg1* reporter expression is confined to the atrial cardiomyocytes and overlaps with Nr2f1a. We have amended our description of the expression in the text (lines 152-154), revised Fig. 1 and provided a new supplemental figure with all the individual channels separated out (S4 Fig.). Additionally, please see our response to Reviewer #2, comment 1, as they brought up a similar issue.

2. The expansion of reg1:GFP into the ventricle region is very clear in TCF site-deleted and tcf7l1a MO embryos in Fig 2C-I. However, it also appears that the intensity of atrial expression of reg1:GFP may be also increased. It would be helpful to perform some quantification of expression level to test if GFP intensity is higher in mTCF, Delta_TCF and tcf7l1a MO embryos.

We have provided quantification of the expression levels via examining intensity of GFP for mTCF and ∆TCF experiments, and the transgenic *3’reg1* embryos depleted for Tcf7l1a. We find the intensity of GFP is increased in these images relative to controls supporting that the expression of GFP is higher in these conditions, as well as having more broad expression throughout that heart. This data has been added to the Results (lines 185) and Fig. 2 of the revised manuscript.

3. In many experiments there is a discrepancy between the treatment effect on enhancer reporter expression and the endogenous nr2f1a expression. For example, BIO treatment resulted in ectopic expansion of reg1:GFP expression into the ventricle (Fig. 3B). If this 3’ enhancer is responsible for restricting nr2f1a expression into the ventricle, then a similar effect would be expected on nr2f1a expression. However, a different effect, an increase in the atrial cardiomyocyte numbers, is observed, based on nr2f1a immunostaining analysis (Fig. 3F). Similar discrepancies are apparent in many other experiments (Fig. 4, for example). It is unclear why the treatments would result in a different effect on the reporter and endogenous gene expression. It would be helpful for the authors to consider two possible explanations: a) Perform HCR / FISH analysis for endogenous nr2f1a mRNA expression in BIO, XAV or foxf1 mRNA injected embryos. It is possible that nr2f1a mRNA expression could be expanded into the atrium in BIO treated or foxf1 mRNA injected embryos but this is not apparent by immunostaining due to other posttranscriptional mechanisms involved in restricting protein localization. b) Quantify fluorescence intensity / expression level of atrial nr2f1a expression (either protein, or even better, mRNA), and 3’reg1:GFP expression intensity in the atrium. Based on the provided images it appears that the atrial reg1:GFP fluorescence is higher in BIO treated or foxf1 mRNA injected embryos, and lower in XAV or dnFoxf1 mRNA injected embryos. It also appears that Nr2f1a immunostaining intensity may follow also a similar pattern, being higher in BIO or Foxf1 mRNA injected embryos, and lower in XAV treated or dnFoxf1 mRNA injected embryos. If this is true, then Wnt inhibition / activation or foxf1 could affect reg1:GFP and endogenous Nr2f1a expression in a similar way, by regulating its expression level in the atrium. In my opinion, this discrepancy is currently the major limitation of this study.

We agree that there are some discrepancies between the *3’reg1:GFP* reporter and endogenous Nr2f1a, which we tried to make clear in the manuscript. It is not unusual that a transgenic reporter for an enhancer may not recapitulate all the properties of a given gene’s expression. Endogenous gene expression can be controlled by interactions between multiple cis-regulatory elements. Therefore, we do not think that some difference between the reporter and endogenous Nr2f1a undermines our interpretations regarding inputs from Wnt signaling and Foxf. Certainly, information from other cis-regulatory elements that control Nr2f1a my help to restrict endogenous expression to the atrium even with increased Wnt signaling and Foxf1, as we point out in the discussion (lines 408-416).

We appreciate Reviewer #3s comment, which is similar to Reviewer #4’s comment 1, suggesting that levels of the reporter and endogenous Nr2f1a may be changed within cardiomyocytes, even if spatial changes are not as obvious as the reporter. While we did mention that Nr2f1a levels may be decreased in XAV treatments of our original manuscript, we did not quantify intensity levels in our images. To address this comment, we have now quantified the intensity of fluorescence in images for *3’reg1:*GFP and Nr2f1a immunohistochemistry within the hearts. Consistent with what was suggested from the representative images, the quantification indicates that with BIO treatment and Foxf1 mRNA injection the intensity of both the GFP reporter and endogenous Nr2f1a is increased, while with XAV treatment and the *dnFoxf1* mRNA injections the intensity was decreased. Additionally, in re-examining our images for this analysis, we find that Tcf7l1a depletion and BIO treatment can cause missexpression of Nr2f1a in some ventricular cardiomyocytes at lower levels, although the ectopic expression is not nearly as dramatic as with the *3’reg1:GFP* reporter. We did not find ectopic expression or low levels of increased Nr2f1a within the ventricular cardiomyocytes following Foxf1 mRNA injection. Therefore, with respect to endogenous Nr2f1a, it appears that expression is increased with BIO and Foxf1 mRNA injection in the atria, even though expression is primarily restricted to the atrial cardiomyoctes. The experiments that we have include intensity measurements in the revised manuscript are Fig. 2, Fig. 3, Fig. 4, Fig. 6, S6 Fig, S13 Fig., and S20 Fig.

We attempted to examine *nr2f1a* mRNA localization with HCR, as was suggested. However, unfortunately, even though we extended our time for resubmission, we were not able to ascertain suitable images of *nr2f1a* expression within the heart with HCR to be able to address the comment regarding examination of localization of *nr2f1a* transcripts. However, we performed RT-qPCR on hearts that were isolated at 24 hpf following the treatments or injection. The RT-qPCR suggests that BIO and FoxF1 mRNA promote, and XAV and dnFoxF1 reduce *nr2f1a* expression within the heart. Taken together with the IHC intensity measures, these results would support that the changes Nr2f1a are accompanied by changes at the transcript level. These experiments are mentioned on lines 227-230 and 276-281 and reported in S7 Fig. of the revised manuscript.

4. The authors argue that foxf1 is upstream of Wnt signaling, in part, because foxf1 mRNA promoted a modest increase in the expression of axin1 and decrease in dkk1a expression. However, this qPCR analysis was performed using whole embryos, therefore it is unclear if the same effect was observed in the heart or atrium. It would be helpful to examine the effect on Wnt signaling in foxf1 overexpressing and / or foxf1 inhibited embryos using Wnt reporter line. Alternatively, HCR / FISH approaches could be used to stain and quantify the level of wnt reporter genes in the heart.

To address this issue, we have repeated the RT-qPCR of Wnt target genes on isolated hearts at 24 hpf following *Foxf1* mRNA injection. This shows that both *axin1* and *axin2* expression are increased, while *dkk1a* is decreased within the heart. In addition, we examined expression of the *7xTcf.Xla.Sia:GFP* Wnt reporter following *FoxF1* and *dnFoxf1* mRNA injection. While we have found that this reporter is not visibly expressed in the heart until after 36 hpf (Moro et al, 2012), we found the Foxf1 mRNA appears to broadly increase reporter expression and in the heart, while dnFoxf1, appeared to decrease the expression within the embryos. Therefore, these data are consistent with Foxf1 causing an increase of Wnt signaling within the embryos. These data were added to the results (lines 335-346) and included in S17 Fig. and S18 Fig. of the revised manuscript.

5. It would be helpful to quantify atrial nr2f1a expression level (HCR / FISH or immunostaining) in 3’reg1 enhancer deleted embryos. nr2f1a expression level could be altered without affecting the number of atrial cardiomyocytes.

We have quantified the levels of Nr2f1a in the *3’reg1* mutant hearts following immunohistochemistry, as mentioned above. Consistent with the suggestion, these would support that the levels of Nr2f1a are decreased even though the total number of Nr2f1a+ cardiomyocytes appears to be unaffected. These data are now mentioned in the results (lines 367-371) and reported in S20 Fig.

**Reviewer #4**: The study by Coppola et. al. is focused characterizing the regulatory elements that drive expression of the nr2f1a, a transcription factor that is important for heart development. This study focuses on the cis-regulatory elements (CRE) that induce expression in atrial cardiomyocytes (AC) in zebrafish. The authors initially determined that the nr2f1a promoter is not sufficient to regulate GFP reporter expression in the atria. This led them to compare ATAC-seq data from several species to identity open chromatin domains. At least 3 open regions were identified at the 3’ end of nr2f1a gene locus and they carefully determined that one specific region, 3’reg1, was sufficient to regulate GFP expression in AC. The 3’reg1 CRE contains conserved binding sites for tcf/lef1, foxf1 and nuclear hormone receptor (NHR) transcription factors. The authors revealed that both Wnt signaling and a Forkhead transcription factor, Foxf1 play a role in regulating nr2f1a expression through the 3’reg1 CRE, while the NHR domain was not required. The authors determine that the Wnt pathway functions downstream or at least in parallel with foxf1 in regulating nr2f1a expression in ACs closest to the venous pole.

To support these findings in functional studies, the authors showed that ectopic expression of Foxf1 from mouse or activation of Wnt signaling was sufficient to expand the AC population without disturbing the ventricle cardiomyocyte population (VC). These observations were supported by blocking foxf1 activity with dominant negative foxf1 and blocking Wnt activity resulted in decreased AC populations.

The authors also addressed whether the absence of foxf1 is required for regulating nr2f1a expression in the heart via the 3’reg1. Here loss-of-foxf1 in F0 embryos showed that GFP fluorescence is decreased. To formally show that nr2f1a expression in the atrial requires 3’reg1, the authors deleted this CRE. In this situation, 3’reg1 deletion did not result in heart defects, but activation of Wnt or injection of Foxf1 mRNA did not expand AC populations. The authors conclude that the 3’reg1 CRE is evolutionary conserved and serves as an important regulator of nr2f1a expression in the atria that is dependent on Wnt and foxf1 activity.

The authors could solidify these studies with these suggestions.

1. Correlating nr2f1a protein expression and transgene reporter is not characterized. There seems to be a discrepancy of nr2f1a protein expression and ectopic GFP expression, particularly in the ventricle. For example, in Figure 3 F, & G there appears to be expression of Nr2f1 protein throughout the atrial, but it is not stated if Nr2f1 is induced in the ventricle as this was not scored. The authors should determine if treatment with BIO not only increase Nr2f1a positive cells in the atrial but are there ectopic expression of the protein in the ventricle.

Please see our response to Reviewer #3, comment 3, as this addresses the same point. Upon closer examination, we do observe some small number of ectopic Nr2f1a+ cardiomyocytes within the ventricle following Tcf7l1a depletion and BIO treatment. However, the number is not close to the ectopic expression of the reporter that can be induced. Additionally, we do not observe this ectopic expression with Foxf1 induction. We have quantified the expression of Nr2f1a within the ventricle with intensity measurements, which also shows a low level of induction. We have modified our interpretations to be consistent with this new data, which is reported in Fig. 3, Fig. 4, and S6 Fig..

2. This could also be extended to the injection of Foxf1 mRNA in Figure 4I. Although the number of AC increased, there was no decrease in VCs. Is it known if there is increased proliferation of only AC cells in these injected conditions? Or is there expansion of cardiogenic domains earlier and this only expanded atrial progenitors? The authors should look earlier for expansion of ACs and determine if there is an increase in proliferation in the atria.

We have examined proliferation by performing EdU labeling at 24 hpf following Foxf1 mRNA injection and BIO treatment. We did not find an effect at this stage on proliferation of atrial cardiomyocytes, suggesting that neither Foxf1 and BIO are promoting an increase in atrial cardiomyocytes via increasing proliferation of progenitor cells or cardiomyocytes. These experiments are referenced on lines 237-241 and 284-285 have been in S9 Fig. of the revised manuscript.

Additionally, we examined *amhc* expression in these embryos at the 22 somite stage and did not observe any difference. Unfortunately, we are not aware of earlier specific atrial progenitor markers that could be used to examine the progenitor population. Thus, we presently favor the interpretation that these signals are promoting expression via affecting the Nr2f1a expression and the differentiation of cells within a progenitor population, which would be similar to models about the temporal effects of differentiation on posterior second heart progenitors from the Moskowitz lab.

3. Knockdown of tcf7l1a expand GFP expression (Figure 2H) in the ventricle but also increase Nr2f1a protein in the ventricle?

We examined expression of Nr2f1a within the ventricle of Tcf7l1a KD embryos. Similar to BIO treatments, we found that there were a few cells that express Nr2f1a in some embryos, though the induction was not nearly a broad as the induction of the reporter. We reference these data in the results (lines 194-204) and present this in Fig. 3 of the revised manuscript.

4. Foxf1 crispants or morpholinos should be used as another assay for loss of foxf1 activity. The use of dominant negative that involves engrailed repressor can sometimes cause a gain of function phenotype that is independent of endogenous foxf1 activity. The authors should also cite original studies that used Engrailed Repressor domain to create dominant negatives.

In the original manuscript, we included crispant data for zebrafish *foxf1* in S6 Fig. (now S12 Fig. of the revised manuscript). These experiments are referenced on lines 272-274 of the revised manuscript. It is not clear from the comments what additional analysis you wanted us to perform.

We have included a citation (Vickers et al, 2002) describing original studies that used engrailed repressor domains to create dominant negative transcription factors (lines 555-556).
